# The association of eating disorder specific and unspecific symptoms with suicidal ideation in patients with anorexia nervosa

**DOI:** 10.1016/j.ijchp.2025.100633

**Published:** 2025-10-16

**Authors:** Evelina Marie Stender, René Freichel, Arne Doose, Inger Hellerhoff, Clara Marie Breier, Ute Lewitzka, Klara Schacht, Daniel Geisler, Joseph A. King, Veit Rössner, Stefan Ehrlich

**Affiliations:** a Translational Developmental Neuroscience Section, Division of Psychological and Social Medicine and Developmental Neurosciences, Faculty of Medicine, Technische Universität Dresden, Dresden, Germany; b University of Amsterdam, Department of Psychology, the Netherlands; c Department of Psychiatry, Psychosomatic und Psychotherapy, Universitätsklinikum Frankfurt - Goethe Universität Heinrich-Hoffmann-Str. 10, 60528 Frankfurt am Main, Germany; d Department of Child and Adolescent Psychiatry and Psychotherapy, Faculty of Medicine, Technische Universität Dresden, Dresden, Germany; e Eating Disorder Research and Treatment Center, Department of Child and Adolescent Psychiatry, Faculty of Medicine, Technische Universität Dresden, Dresden, Germany

**Keywords:** Anorexia nervosa, Suicidality, Suicidal ideation, Females, Network analysis

## Abstract

Anorexia nervosa is a serious eating disorder with a high mortality rate, ranking among the most lethal mental health conditions. This is not only due to sequelae of cachexia, but also due to suicidality. The present study employs a network analysis approach to determine whether there are unique associations between suicidal ideation and eating disorder-specific symptoms in anorexia nervosa, or if suicidal ideation is more influenced by other symptoms such as those more loosely related to eating disorders or general internalizing symptoms, as well as biological factors. Additionally, we examined the potentially changing impact of symptoms after intensive treatment. *Methods:* The study involved female patients with anorexia nervosa admitted to intensive inpatient treatment. Eating disorder-specific and -related symptoms, depressive, anxiety, obsessive-compulsive symptoms, nutritional data as well as suicidal ideation were assessed at two points: immediately after admission (*n* = 313) and following partial weight gain (*n* = 217) and were examined together in a network analysis model. *Results:* The nodes that were most strongly related to suicidal ideation at both timepoints were feelings of ineffectiveness, as well as depressive and anxiety symptoms. Eating disorder-specific symptoms were found to be not significantly related to suicidal ideation. *Conclusions:* The results indicate that suicidal ideation in anorexia nervosa is primarily related to broader psychological symptoms rather than eating disorder-specific symptoms. The prominence of feelings of ineffectiveness within the network highlights the need for clinical interventions that focus on the enhancement of self-efficacy in anorexia nervosa.

## Introduction

Anorexia nervosa (AN) is an eating disorder (ED) characterized by an extreme restriction of caloric intake and weight loss, leading to severe underweight (American Psychiatric Association & American Psychiatric Association, 2013). It occurs predominantly in females with an onset during adolescence or young adulthood (Zipfel et al., 2015). Lifetime prevalence ranges from 0.1 % to 3.6 % (Van Eeden et al., 2021). In addition to the symptoms typically associated with EDs, AN is also characterized by a range of other internalizing difficulties, including depressive, anxiety, and obsessive-compulsive symptoms (Jagielska & Kacperska, 2017).

Suicidality is quite common in AN. In a meta-review, AN stands out as one of the mental disorders with the highest mortality rates alongside substance use disorders (e.g. opioids) and has one of the highest suicide risks besides borderline personality disorder, depression and bipolar disorder (Chesney et al., 2014). Among the causes of death associated with AN, suicide ranks second in frequency, surpassed only by medical complications directly related to the disease (Arcelus et al., 2011; Papadopoulos et al., 2009). Not only completed suicides, but also suicide attempts (3–20 %) (Franko & Keel, 2006) as well as suicidal ideation (SI; 20–43 %, particularly 19.8 % for AN restrictive subtype) (Arnold et al., 2023; Smith et al., 2018b) occur more often in AN than in the general population.

A question of utmost clinical relevance is: What factors are associated with SI in AN?

Solmi et al. (2018) examined an AN-network including ED-specific, ED-related and internalizing features, and a nutritional marker (BMI), identifying ED-specific symptoms (drive for thinness), depression and anxiety, and ineffectiveness as central to the disorder. Expanding the focus to SI, Smith et al. (2018a) propose a possible bidirectional – both direct and indirect – relationship between ED-symptoms and suicidality in their review paper. Supporting this, severity of ED-symptoms (measured via composite scores on ED-specific standardized interviews or questionnaires) has been linked to SI (Bühren et al., 2014; Mereu et al., 2022). Smith et al. (2018a) further emphasize the relevance of shared mechanisms – like co-occurring psychological symptoms, biological markers and ED-related psychological constructs (e.g. interoceptive deficits) – as contributing to both suicidality and EDs. Building on these theoretical considerations and empirical findings as a conceptual framework, the present analysis includes ED-specific, ED-related, (co-occurring) general internalizing symptoms and a nutritional indicator to examine their interplay in SI in AN.

To differentiate between the two ED-dimensions, we use the Eating Disorder Inventory-2 (EDI-2), which distinguishes between ED-specific (EDI-2-subscales drive for thinness, bulimia, body dissatisfaction) and ED-related symptoms (all other EDI-2 subscales such as perfectionism, ineffectiveness, interoceptive awareness) (Nevonen & Broberg, 2001). Regarding the link between ED-specific symptoms and suicidality, the evidence is mixed. Based on the Interpersonal Theory of Suicide (IPTS; Joiner et al., 2005), Selby et al. (2010) propose two potential pathways to suicidality in AN: one through repetitive compensatory behaviors in the AN binge-purge subtype (conceptually comparable to EDI-2 bulimia subscale) and another through starvation resulting from restrictive behavior in the AN restrictive subtype (conceptually reflected in the EDI-2 drive for thinness subscale). Supporting this, purging – but not binging – predicted SI in a mixed ED-sample (Joiner et al., 2022). In contrast, restrictive behaviors – but not binge/purge symptoms – predicted SI in low-weight ED patients (Wang et al., 2019). Body dissatisfaction, a construct related to drive for thinness (Faust, 1987), has also been linked to SI both in AN (Mereu et al., 2022; Stein et al., 2003) and a non-clinical adolescent sample (Brausch & Perkins, 2018), potentially through increased perceived burdensomeness (Forrest et al., 2016).

Research on ED-related symptoms and their relationship to SI is more limited. Factors such as low self-esteem, interpersonal insecurity, and interoceptive deficits have been linked with SI in AN (Mereu et al., 2022). Interoceptive deficits have also been linked to SI (Ortiz & Smith, 2019) in mixed ED-samples as well as in an online study (Forrest, Smith, White and Joiner, 2015). Taken together, these findings suggest that not only ED-specific, but also ED-related symptoms might play a central role in SI in AN.

In addition to ED-specific and ED-related symptoms, general internalizing symptoms including depressive, anxiety and obsessive-compulsive symptoms can also significantly influence SI in AN (Smith et al., 2018b). In particular, affective and anxiety symptoms, which are quite common in AN-patients, contribute to SI according to some literature reviews (Dancyger & Fornari, 2005). Some results even suggest an attenuation or disappearance of the statistical relationship between ED-specific symptoms and suicidality when general internalizing symptoms were considered (Bodell et al., 2013).

Apart from psychological symptoms, biological factors like genetic predispositions or endocrine signals might also contribute to SI in AN (Lengvenyte et al., 2021; Thornton et al., 2016). Dysregulated gene expression affecting stress response, immune function, serotonergic and dopaminergic neurotransmission, as well as neuroplasticity has been linked to increased suicide vulnerability (Lengvenyte et al., 2021). In AN, cachexia and low peripheral levels of the adipokine leptin, i.e. hypoleptinemia – a marker of acute underweight, undernutrition and hypometabolism (Hebebrand et al., 2022) – are associated with affective symptoms and rumination (Ehrlich, Burghardt, Schneider, Hein et al., 2009; Fürtjes et al., 2018) as well as grey matter reductions in hippocampal areas and the amygdala (Bahnsen et al., 2024; Wronski et al., 2022, 2023). Moreover, leptin levels have been linked to SI in other EDs (Musial et al., 2025; Smith et al., 2018b; Wade et al., 2015) and to symptom change in major depressive disorder (Esel et al., 2005).

As adolescence is a critical developmental period for the onset of EDs and self-harming behaviors (Campbell & Peebles, 2014; Gvion & Fachler, 2017), age might play a role, moderating SI in AN (Goldstein & Gvion, 2019). Few studies have directly examined age as a risk factor for suicidality in AN. Looking at SI in the general population, two representative studies of adolescents and adults found that SI was significantly more common in younger age groups (Cooper et al., 2015; Thompson et al., 2011).

Despite evidence implicating ED-specific, ED-related, internalizing symptoms and biological factors in SI in AN, it remains unclear how these domains interact. Prior studies largely relied on latent variable or regression models that assume unidirectional relationships and treat symptoms as interchangeable indicators of a latent construct (e.g. AN) (Cramer et al., 2010). These models overlook clinical reality: (1) a single latent construct (e.g. AN) likely cannot account for all symptoms (Levinson et al., 2022) or SI co-occurrence; (2) symptoms are not independent of their causes and often influence each other through feedback loops (Borsboom, 2017; Borsboom & Cramer, 2013); and (3) they provide limited guidance for targeted interventions (Burger et al., 2020; Levinson et al., 2022). Moreover, they also fail to explain important ED characteristics such as comorbid symptoms and heterogeneity, and cannot distinguish direct from indirect effects or model synergistic interactions (Cramer et al., 2010). Network analysis offers a framework that captures these complexities by modeling psychopathology as dynamic systems of interacting symptoms (Borsboom, 2017; Bringmann & Eronen, 2018). It reveals central symptoms —those most influential in the system—and potentially the most effective targets for intervention while controlling for the rest of the system (Bringmann & Eronen, 2018; Borsboom & Cramer, 2013; Fried et al., 2016; Levinson et al., 2022). It also aligns with cognitive-behavioral therapy’s symptom-level focus (Beck, 2011, Wenzel, 2012). Network approaches thus provide a powerful alternative to traditional models, yielding mechanistic and clinically actionable insights (as central nodes or edges) for prevention and treatment (Jones et al., 2021; Levinson et al., 2023).

To date, no known study focused on AN has investigated suicidality using a network analytic between-person approach. However, a recent published proof-of-concept study illustrated how a network analytic approach on a within-person level (based on longitudinal diary data) can be implemented to personalize the treatment for patients with AN at an elevated risk for suicide (Harris et al., 2025). Using a between-person approach in a network analysis of data collected from a mixed ED-sample, the results indicated that interoceptive difficulties served as a connection between suicidality and ED-symptoms (Smith et al., 2020). Additionally, SI was identified as bridge symptom between ED and a depressive symptom cluster in non-clinical samples (Wu et al., 2023; Yang et al., 2023). While applying the conceptual and methodological framework of Solmi et al. (2018) specifically to SI, the present study aims to build upon and extend these previous findings in several important ways. First, we focus exclusively on individuals with AN, allowing for a more targeted investigation within this diagnostically and clinically distinct population. Second, by incorporating both longitudinal and nutritional data (BMI, leptin), we offer a more comprehensive and temporally sensitive perspective. Most importantly, our study explicitly differentiates between ED-specific, ED-related and (co-occurring) general internalizing symptoms (e.g., depression, anxiety, obsessive-compulsive symptoms) within the network, enabling us to identify the domains most closely associated with SI. This analysis may identify potential starting points for effective therapeutic interventions and advances personalized risk assessments.

## Methods

### Participants

A total of 313 acutely underweight (see BMI criteria and criteria for the assessment timepoint with regards to treatment initiation below) female adolescents or young adults (aged 12–29 years) with AN participated in this study. Patients were admitted to intensive inpatient treatment of a specialized ED program at a child and adolescent psychiatry or psychosomatics department of a university hospital and assessed within 4 days (weight, height, leptin measures) or, respectively, 10 days (questionnaires) after admission to inpatient treatment, i.e. after the beginning of a behaviorally orientated nutritional rehabilitation program (timepoint 1, T1). A total of 217 patients with AN were reassessed after short-term weight restoration (timepoint 2, T2; body mass index (BMI) increase of at least 10 % was an inclusion criterion). Reasons for dropout included insufficient weight gain, premature discharge, and withdrawal of consent.

Current AN, according to DSM-5 criteria, was diagnosed using a modified version of the expert form of the Structured Interview for Anorexia and Bulimia Nervosa (SIAB-EX; Fichter & Quadflieg, 1999) and required a BMI below the 10th age percentile (if <15.5 years of age) or below 17.5 kg/m2 (if >15.5 years of age). Information regarding exclusion criteria was obtained from all participants using the SIAB-EX supplemented by our own semi-structured interview and medical records (see supplemental material (SM) S1). Comorbid diagnoses were taken from medical records and confirmed by an expert clinician. Participants were excluded if they had any of the following clinical diagnoses: organic brain syndrome, schizophrenia, substance abuse/dependence, psychosis NOS, bipolar disorder, bulimia nervosa or binge-eating disorder. Further exclusion criteria were IQ lower than 85, psychotropic medication within 4 weeks prior to the study (except medication with olanzapine (*n* = 4) and SSRI (*n* = 12), which was allowed), current inflammatory, neurologic or metabolic illness, chronic medical or neurological illness that could affect appetite, eating behaviour, or body weight (e.g., diabetes), clinically relevant anemia, pregnancy and breast feeding.

Study data were collected and managed using secure, web-based electronic data capture tools REDCap (Research Electronic Data Capture; P. A. Harris et al., 2009). This study was approved by the local Institutional Review Board, and all participants (and if underage their guardians) gave written informed consent.

### Clinical measures

All clinical measures at T1 were assessed within the first ten days after study inclusion. The EDI-2 (Thiel et al., 1997) was used to assess both ED-specific symptoms (including the subscales drive for thinness, bulimia, and body dissatisfaction) and psychological characteristics only loosely related to disordered eating (in this paper referred to as ED-related symptoms: subscales ineffectiveness, perfectionism, interpersonal distrust, interoceptive awareness, and maturity fears) (Nevonen & Broberg, 2001). General internalizing symptoms, i.e. depressive, anxiety and obsessive-compulsive symptoms were assessed using the respective scales of the Symptom Checklist-90-Revised (SCL-90-R; Franke & Derogatis, 2002). Moreover, item #9 (suicidal thoughts or wishes – ranging from 0 (“I don't have any thoughts of killing myself.”) to 4 (“I would kill myself if I had the chance.”)) from the Beck Depression Inventory-II (BDI-II; Hautzinger et al., 2009) was used. BMI standard deviation scores (BMI-SDS; Hemmelmann et al., 2010; Kromeyer-Hauschild et al., 2001) were computed to provide an age-corrected index. We estimated IQ using short versions of the Wechsler Adult Intelligence Scale (von Aster et al., 2006) or the Wechsler Intelligence Scale for Children (Petermann & Petermann, 2010). For plasma leptin level measurements see SM S2.

### Statistical analyses

All analyses were conducted using R, version 4.3.2 (RStudio Team, 2023). If not indicated otherwise, all values are presented as mean±standard deviation. In terms of descriptive statistics, histograms and Shapiro-Wilk-tests were employed to test the underlying statistical assumptions. All variables were tested for significant differences between T1 and T2.

Missing data ranged between 0 % and 14.7 % (mean: 8.2 %) per variable and was imputed using the R-package *mice* (Van Buuren & Groothuis-Oudshoorn, 2011), version 3.16.0. Data was imputed if no >12 out of the 23 variables necessary for the network analysis were missing per participant (SCL-90-R-items for generating depression score, items for generating SI score, EDI-2-subscales, SCL-90-R subscales for anxiety and obsessive-compulsive symptoms).

Two scores, a depression and a SI score, were computed. The depression score comprised all SCL-90-R items, assigned to the depression subscale, excluding item #15 (as it was used for generating the SI score). This sum score was computed in accordance with the SCL-90-R manual (Franke & Derogatis, 2002). To obtain an overall measure of SI (SI score), we conducted a factor analysis on the three items that measured specific dimensions of SI as suicidal thoughts (using SCL-90-R items #15 (thoughts of ending one’s life) and #59 (thoughts of death or dying)), along with BDI-II item #9 (suicidal thoughts and wishes). Items were z-transformed and weighted based on their factor loadings (SCL-90-R question_15: 0.91, SCL-90-R question_59: 0.90, BDI-2 question_9: 0.73). Evidence of high internal consistency, alongside convergent and discriminant validity, provides psychometric support for the SI node. For further details please refer to SM S3.

We modeled symptoms as a network in which nodes represent individual variables (e.g., symptoms or subscales), and edges are weighted by regularized partial correlation coefficients (denoted by *r_par_*), reflecting the strength of the direct association between variable pairs after controlling for all others. Visually, the thickness and color saturation of the edges reflect the strength of the regularized partial correlation coefficients. Positive partial associations are depicted by blue edges, while negative ones are shown in red. The network comprised twelve nodes: the SI score, all eight EDI-2 subscales, and the three SCL-90-R subscales – depressive, anxiety, and obsessive-compulsive symptoms. Symptom networks were created for T1 (*n* = 313) and T2 (*n* = 217). A supplementary analysis, integrating leptin into the networks (n_T1_=229, n_T2_=150) was conducted and is described in the SM S5. To control for potential effects of psychoactive medication or age on the network, we conducted a sensitivity analysis to examine the potential confounding effects of psychoactive medication (*n* = 15) and age. Thus, we re-estimated the networks including only those participants without psychoactive medication (n_T1_=298, n_T2_=208) or below the age of 18 (n_T1_=253, n_T2_=183) or – respectively – added age as node to the network. Additionally, to control for potential influencing effects due to the number of nodes in the ED-symptom cluster, we ran additional analyses, including only ED-specific symptoms (bulimia, body dissatisfaction, drive for thinness) or only ED-related symptoms (ineffectiveness, interoception, perfectionism, interpersonal distrust, maturity fears).

Partial correlation networks were estimated using the *bootnet*-package (Epskamp et al., 2018) and visualized using the *qgraph*-package (Epskamp et al., 2012), version 1.9.5. The network model employs the “EBICglasso” algorithm, which implements the "least absolute shrinkage and selection operator" (LASSO) regularization using the extended Bayesian information criterion (EBIC) for model selection. To achieve a sparse and interpretable network, we set the tuning parameter to 0.5. This choice was considered appropriate as it balances sensitivity and specificity with respect to the existence of an edge and is a commonly used approach (Freichel, 2023).

Centrality indices, specifically node strength and expected influence (EI), were computed to assess the importance of nodes in the network. Node strength is defined as the sum of the absolute edge weights (*r_par_*) of all edges connected to a node, and is one of the most commonly reported metrics due to its stability and interpretability (Bringmann et al., 2019). EI, by contrast, retains the sign of edge weights and is defined as the sum of all signed *r_par_* values for edges connected to a node. To further examine centrality across symptom clusters, bridge expected influence (bridge EI) was calculated, defined as the sum of all signed *r_par_* values connecting a given node to nodes outside its own cluster. The stability of the network was estimated following the recommendations by Epskamp et al. (2018). Correlation stability (CS) coefficients were calculated for all networks, representing the maximum proportion of the population that can be omitted while maintaining a correlation of at least 0.7 between the newly calculated indices and those of the original network. A minimum cut-off value of 0.25 indicates acceptable network stability (Epskamp & Fried, 2018). Additionally, non-parametric bootstrapping (Bollen & Stine, 1992) was employed to estimate the 95 % confidence intervals around the estimated edge weights, providing insights into edge accuracy.

**Table 1:** Study sample: demographic and clinical characteristics.

	T1	T2	significant differences
Age, years	15.89±2.29	16.15±2.28	
BMI, kg/m^2^	14.66±1.32	18.84±1.37	T2 > T1***
BMI-SDS	−3.16±1.08	−0.73±0.69	T2 > T1***
Leptin, µg/L	−0.18±0.76	1.01±0.33	T2 > T1***
EDI-2 total	212.77±43.98	202.21±47.92	T2 < T1***
SI	0.44±0.56	0.41±0.6	not significant
SCL-90-R DEPR	2.6 ± 0.86	2.03±0.81	T2 < T1***
SCL-90-R ANX	0.91±0.75	0.74±0.74	T2 < T1**
SCL-90-R OCS	1.16±0.82	0.78±0.66	T2 < T1***

Note. Mean values±standard deviation for each variable are shown separately for each time point. Differences were tested using Wilcoxon signed-rank test (**p < 0.01, ***p < 0.001).

Within the AN group, 267 (85 %) of the patients were of the restrictive, 36 (12 %) of the binge/purging subtype and 10 (3 %) patients have not been assigned to one subtype. The mean age of illness onset was 14.3 ± 2.5 years and the mean duration of the current illness in the AN group was 13.7 ± 17.7 months. 67 (22 %) had comorbid mental disorders (11 % depressive disorders including dysthymia (*n* = 35), 5 % obsessive compulsive disorder (*n* = 17), 8 % anxiety disorders (*n* = 24), 1 % Post-traumatic stress disorder (*n* = 3), 0.7 % tics/Tourette Syndrome (*n* = 2), 0.7 % adjustment disorder (*n* = 2), 0.3 % conduct disorder (*n* = 1), 0.3 % developmental disorders (*n* = 1) and 1 % showed hints for personality disorders (*n* = 3)). IQ-results were on average at 110±12.6. At T1, 15 patients were on psychoactive medication (SSRI (*n* = 12) or olanzapine (*n* = 4)).

Abbreviations: T1, acute anorexia nervosa (AN) participants at timepoint 1 (admission); T2, AN participants at timepoint 2 (assessment following an increase in BMI of 10 % or more); BMI, body mass index; BMI-SDS, body mass index standard deviation score; EDI-2 total, Eating Disorder Inventory-2, total score; SI, suicidal ideation as measured via BDI-II (Beck Depression Inventory-II) Item #9; SCL-90-R, Symptom Checklist-90-Revised.; DEPR, depressive symptom score of SCL-90-R (generated without item #15); ANX, anxiety symptom score of SCL-90-R; OCS, obsessive-compulsive symptom score of SCL-90-R.

To investigate potential differences in the networks between timepoints, network comparison tests (NCTs) were conducted using the R-package *NetworkComparisonTest*, version 2.2.2 (Van Borkulo et al., 2023). NCTs involved comparing T1 networks against T2 networks with 1000 permutations to assess both differences in network strength and structure. Furthermore, the differences in the strength of each edge between the two networks were evaluated, both with and without using the Bonferroni-Holm criterion for multiple comparisons.

## Results

### Sample characteristics

Tables 1 and S1 provide a summary of demographic and clinical characteristics. All participants were of female sex and gender. 253 participants (81 %) were below the age of 18 years. Within the AN group, 267 (86 %) of the patients were of the restrictive subtype. The mean age of illness onset was 14.3 ± 2.5 years and the mean duration of illness in the AN group was 13.7 ± 17.7 months. Participants were predominantly White (*n* = 307, 98 %), with a minority identifying as Asian (*n* = 2, 0.6 %) (missing values: *n* = 4). The socio-economic status was 2.09±0.67 (for details see SM S2).

Mean BMI-increase at the follow-up timepoint (T2) was 29.1 %. Not only BMI and BMI-SDS, but also leptin levels were significantly lower at T1 compared to T2. Nearly half of the patients (*n* = 135, ∼45 %) reported SI (i.e. thoughts about killing oneself or a wish to kill oneself) at T1 (*n_thoughts_*=124, ∼41 %; *n_wish_*=11, ∼4 %). After partial weight restoration (T2) a third (*n* = 77, ∼36 %) reported SI (*n_thoughts_*=68, ∼32 %; *n_whish_*=9, ∼4 %; refer to Table 2 for further details). EDI total score, most EDI-subscales (such as drive for thinness, bulimia, ineffectiveness, perfectionism, interoceptive awareness, and maturity fears) and general internalizing symptoms scores (such as depressive or anxiety symptoms) were significantly higher at T1 compared to T2.

**Table 2:** Study Sample: distribution of suicidal ideation at timepoint 1.

Suicidal ideation score	T1		T2	
	n	%	n	%
0 (“I don’t have any thoughts of killing myself.“)	166	55	137	64
1 (“I have thoughts of killing myself, but I would not carry them out.“)	124	41	68	32
2 (“I would like to kill myself.“)	9	3	7	3
3 (“I would kill myself if I had the chance.“)	2	0.7	2	1

Note. Suicidal ideation scores as measured via BDI-II item #9.

### Suicidal ideation network – eating symptoms; depressive, anxiety, and obsessive-compulsive symptoms

**Fig. 1:**
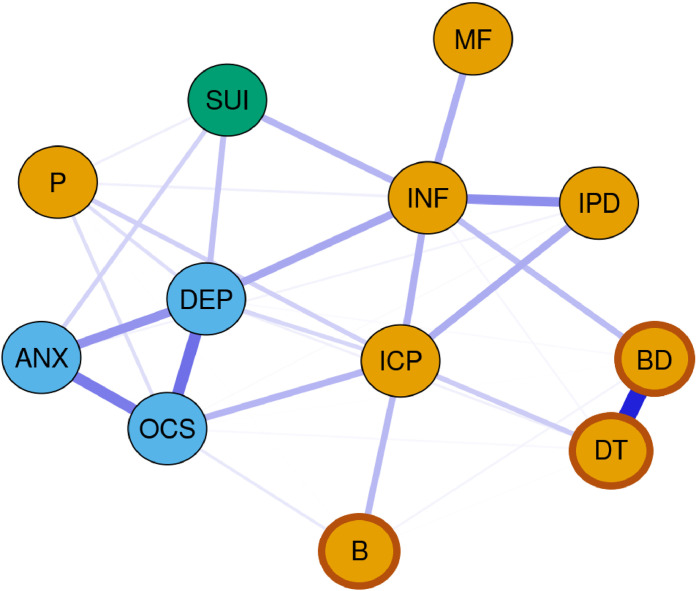
Estimated network of overall patient population (*n* = 313) at timepoint 1. Blue nodes: Subscales of Symptom Checklist-90-Revised. Orange nodes: Subscales of Eating Disorder Inventory-2 (EDI-2). Darker frame around orange nodes: ED-specific symptoms (EDI-2). Blue lines: positive partial associations. Red lines: negative partial associations. Thickness of edges: magnitude of the partial correlation.

Note. SI, suicidal ideation; DEP, depressive symptom score of SCL-90-R (generated without item #15); ANX, anxiety symptom score of SCL-90-R; OCS, obsessive-compulsive symptom score of SCL-90-R; DT, EDI-2 scale drive for thinness; B, EDI-2 scale bulimia; BD, EDI-2 scale body dissatisfaction; INF, EDI-2 scale ineffectiveness; P, EDI-2 scale perfectionism; IPD, EDI-2 scale interpersonal distrust; ICP, EDI-2 scale interoception; MF, EDI-2 scale maturity fears.

**Fig. 2:**
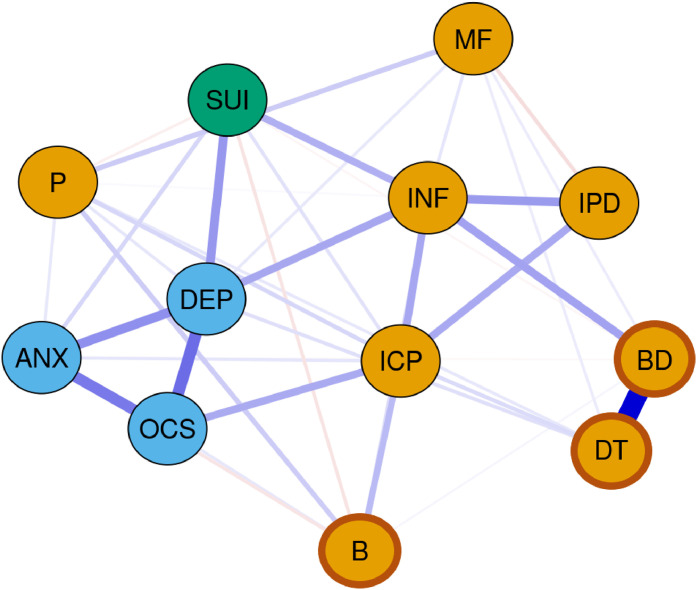
Estimated network of overall patient population (*n* = 217) at timepoint 2. Blue nodes: Subscales of Symptom Checklist-90-Revised. Orange nodes: Subscales of Eating Disorder Inventory-2 (EDI-2). Darker frame around orange nodes: ED-specific symptoms (EDI-2). Blue edges: positive partial associations. Red edges: negative partial associations. Thickness of edges: magnitude of the partial correlation.

Note. SI, suicidal ideation; DEP, depressive symptom score of SCL-90-R (generated without item #15); ANX, anxiety symptom score of SCL-90-R; OCS, obsessive-compulsive symptom score of SCL-90-R; DT, EDI-2 scale drive for thinness; B, EDI-2 scale bulimia; BD, EDI-2 scale body dissatisfaction; INF, EDI-2 scale ineffectiveness; P, EDI-2 scale perfectionism; IPD, EDI-2 scale interpersonal distrust; ICP, EDI-2 scale interoception; MF, EDI-2 scale maturity fears.

**Fig. 3:**
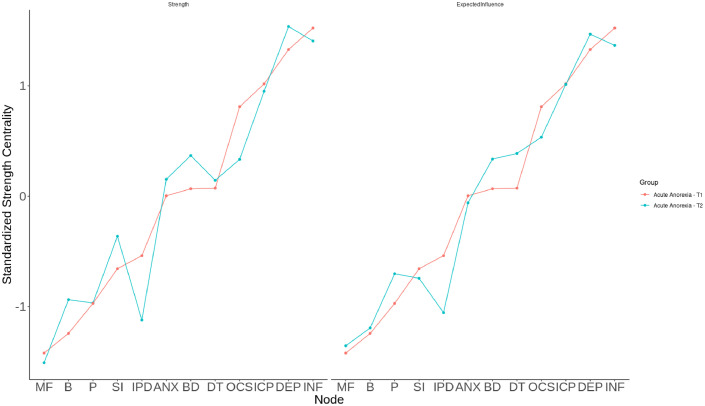
Plotted centrality indices (strength and expected influence) of each node in original networks at timepoint 1 and timepoint 2. Red line: acute anorexia nervosa patients at timepoint 1; blue line: anorexia nervosa patients at timepoint 2.

Note. MF, EDI-2 scale maturity fears; B, EDI-2 scale bulimia; P, EDI-2 scale perfectionism; SI, suicidal ideation; IPD, EDI-2 scale interpersonal distrust; ANX, anxiety symptom score of SCL-90-R; BD, EDI-2 scale body dissatisfaction; DT, EDI-2 scale drive for thinness; OCS, obsessive-compulsive symptom score of SCL-90-R; ICP, EDI-2 scale interoceptive awareness; DEP, depressive symptom score of SCL-90-R (generated without item #15); INF, EDI-2 scale ineffectiveness.

The networks at T1 and T2 are depicted in Fig. 1, Fig. 2. Centrality measures are visualized in Fig. 3. Both networks demonstrate acceptable stability (CS-coefficient strength at T1: 0.75, CS-coefficient strength at T2: 0.75; for plotted edge centrality see SM Figures S1 and S2). In both networks, ineffectiveness (T1: *EI*=1.36, T2: *EI*=1.33), depressive symptoms (T1: *EI*=1.29, T2: *EI*=1.37) and problems with interoceptive awareness (T1: *EI*=1.17, T2: *EI*=1.2) were the nodes with the highest EI in the network. Additionally, bridge EI was highest for depressive symptoms, SI, ineffectiveness and interoceptive awareness (see SM Table S2 and SM Figure S27 for further details).

The general internalizing symptoms (depressive, anxiety, obsessive-compulsive symptoms) as well as some ED-specific symptoms (drive for thinness, body dissatisfaction) were strongly interrelated.

Of particular interest to our research question regarding potential factors associated with SI were the positive connections of SI with ineffectiveness (*r_par_T1_*=0.19, *r_par_T2_*=0.21), depressive symptoms (*r_par_T1_*=0.16, *r_par_T2_*=0.27) and anxiety symptoms (*r_par_T1_*=0.12, *r_par_T2_*=0.11) at T1 and T2. Additionally, at T1 SI shared a much weaker positive edge with perfectionism (*r_par_T1_*=0.04). At T2, the edge with perfectionism turned negative (*r_par_T2_*=−0.04). Moreover, a positive edge with interoceptive awareness (*r_par_T2_*=0.08) as well as negative edges with bulimia (*r_par_T2_*=−0.07) and body dissatisfaction (*r_par_T2_*=−0.03) arose at T2. For further details see SM Tables S3 and S4.

When comparing the networks of the two timepoints using the NCT, a significant difference in global strength, but not network invariance was observed (*S* = 0.87, *P* = 0.04; *M* *=* 0.14, *P* = 0.6, for further detail see SM Table S5). Global strength was higher at T2 compared to T1 (*S_T1_*=4.6, *S_T2_*=5.5), indicating a higher level of connectivity between nodes at T2. When not using p-adjustment methods, a significantly different edge between the two networks with respect to our main research question was SI – perfectionism (*P* = 0.02; for other edges that were different between the two timepoints but not related to our main hypothesis see SM S4).

### Exploratory analyses: potential effects of leptin, age or psychoactive medication on the networks

The generated networks that also consider possible effects of leptin, age or psychoactive medication (*n* = 12 (∼4 %) patients with SSRI, *n* = 4 (∼1 %) patients with olanzapine) can be found in the SM (leptin: Figures S3-S12, age: Figures S13-S18, psychoactive medication: Figures S19-S22). The inclusion of endogenous hormone leptin did not change the results reported above significantly (see SM S5, Tables S6-S8, Figures S3-S12).

The effect of age (included as a node (see SM Figures S13-S16) or addressed by running a supplementary model only based on the data of participants below the age of 18 years (see SM Figures S17-S18)) had no substantial impact on the network regarding the above-mentioned main findings (i.e. ineffectiveness, depressive and anxiety symptoms, but not ED-specific symptoms sharing important edges with SI).

Psychoactive medication also had no substantial impact on the network regarding the above-mentioned finding (demonstrated by rerunning the models in medication free patients *n_T1_*=298, *n_T2_*=208; SM Figures S19-S22). Moreover, limiting the analysis to either only ED-specific symptoms (SM Figures S23-S24) or only ED-related symptoms (SM Figures S25-S26) did not significantly alter the main findings. Regardless of whether these symptom types were examined separately or not, the only nodes showing strong connections with SI were ineffectiveness, anxiety, and depressive symptoms.

## Discussion

The aim of our study was to shed light on the symptoms associated with SI in AN. In our predominantly adolescent sample, SI played an important role in individuals with AN, with about half of the patients experiencing SI at the start of treatment (T1) and still about one-third experiencing suicidal thoughts after about 3 months of intensive treatment (T2). This aligns with previous findings that have reported SI rates ranging from 20 % to 43 %, underscoring the profound impact of SI in this patient group (Smith et al., 2018b). Therefore, it is crucial to implement more intensive interventions at an early stage.

By applying network analysis techniques, we were able to identify three time-invariant, i.e. state-independent factors associated with SI: ineffectiveness as well as depressive and anxiety symptoms. In addition to their strong connections with SI, indicated by the strong edge weights (see Fig. 1, Fig. 2, and SM Tables S3 and S4), these symptoms also emerged as the most central within the SI network and as symptoms that link across the symptom cluster, including connections to SI. While bridge EI does not specify which or how many clusters are connected, the high values suggest that these symptoms link across cluster boundaries. This means they are the nodes with the greatest influence, possibly causing the most changes throughout the entire network when targeted by interventions (Robinaugh et al., 2016). Our results align with a previous network analysis, which identified ineffectiveness, along with depressive and anxiety symptoms, as the most central symptoms in AN (Solmi et al., 2018). Additionally, they extend former results by suggesting that ED-related and general internalizing symptoms, especially affective and anxiety symptoms, determine SI in AN (Dancyger & Fornari, 2005; Franko & Keel, 2006). Depressive symptoms drive suicidality in many different mental health conditions (Esposito & Clum, 2002; Liu et al., 2022; Nock et al., 2009) and suicidality is a core symptom of depressive disorder (Arnone et al., 2024; Wang et al., 2024). Regarding anxiety symptoms our finding is also in line with studies and reviews reporting a link to suicidality, both in individuals with EDs but also in the general population (Cougle et al., 2009; Dancyger & Fornari, 2005; Diefenbach et al., 2009; Kanwar et al., 2013). Furthermore, the meta-analysis by Kanwar et al. (2013) indicated no association of suicidality and Obsessive-Compulsive-Disorder, aligning our finding of no direct link between suicidality and obsessive-compulsive symptoms.

To our knowledge, no study so far has identified an association between ineffectiveness and suicidality in AN. Interestingly, ineffectiveness, an ED-related symptom, had not only high EI values, indicating it to be a potentially promising target of intervention for generating change in the whole network, but also edge weights indicate ineffectiveness to be almost as influential on SI as depressive symptoms. Ineffectiveness as measured via EDI-2 is conceptualized as low self-esteem and low self-efficacy taken together with an emotional component of insufficiency (Thiel et al., 1997). A study in AN reported a higher score of eating-related self-efficacy (i.e. the opposite of ineffectiveness) at admission to treatment to be related to a shorter length of stay at the hospital, lower score on the EDI-2 body dissatisfaction scale, and a higher weekly weight gain after discharge (Pinto et al., 2008). Regarding low self-efficacy, a key component of ineffectiveness, studies examining non-AN samples found an association with SI in psychiatrically hospitalized military personnel (Daruwala et al., 2018) as well as in non-clinical/population-based samples (Kobayashi et al., 2015; Li et al., 2023). Furthermore, to theoretically support this relationship, the IPTS (Joiner, 2005) can be referenced. According to this theory, perceived burdensomeness (which is closely related to ineffectiveness) is considered a key component contributing to suicidal behavior. This hypothesized relationship is well-supported by numerous studies, reviews and meta-analyses (e.g. Chu et al., 2017; Hill & Pettit, 2014). For example, in a very large meta-analysis of predominantly community samples, perceived burdensomeness was found to be moderately and significantly associated with SI (*r* = 0.48, *p* < 0.001; *k* = 84; *N* = 37,894) (Chu et al., 2017). In their literature review, Hill and Pettit (2014) reported significant cross-sectional associations between perceived burdensomeness and suicidality in clinical samples, identifying it as either moderator or mediator between risk and protective factors and suicidal behavior. Since ineffectiveness appeared to be one of the central factors associated with SI in our network-model, it might be a good target for psychotherapeutic interventions. At the onset of inpatient treatment for adolescents with AN, opportunities for patients to actively participate in and influence the treatment process are limited (Mac Donald et al., 2023; Zielinski-Gussen et al., 2023). This lack of involvement may subjectively be perceived as coercive, which, as some studies have indicated, may contribute to an increase in negative affect and consequently suicidality (Mac Donald et al., 2023; Zielinski-Gussen et al., 2023). Our findings could potentially reflect this correlation as well. Therefore, therapeutic methods targeting self-efficacy like shared decision making and empowerment might be especially important for AN-patients suffering from SI and suicidality in general.

In addition to those state-independent factors associated with SI, we also found some time-dependent associations: At admission to treatment, a positive but weak relationship of perfectionism (ED-related symptom) and SI arose. This observed relationship has been previously described in many clinical and non-clinical samples (O’Connor, 2007) and is therefore not surprising. Interestingly, this association shifted to a weak negative connection after partial weight gain, making perfectionism appear as a protecting factor against SI. However, both associations are questionable since the NCT, testing for significant differences of the edges between the initial assessment and after partial weight gain, only became significant when not correcting for multiple testing. Also, in the light of positive edges between (a) perfectionism and depressive symptoms and (b) perfectionism and ineffectiveness and those nodes being positively partially correlated to SI, doubt is cast on a direct negative relationship between perfectionism and SI.

Following partial weight gain, the nodes of the network were more strongly connected compared to T1. In general, variables became more interconnected, which could potentially be leveraged in psychotherapeutic treatment, as it suggests that addressing one aspect could lead to changes in other areas. However, caution is advised when interpreting this finding, as we did not examine directed effects. This increased connectivity is also particularly relevant in relation to SI, as new (albeit weak) edges emerged, indicating that, at T2, more variables exerted an influence on SI. This highlights the growing complexity of the network, particularly with respect to SI, over time. More specifically, some significant but very weak associations emerged with bulimia (negative edge), body dissatisfaction (negative edge) and interoceptive awareness (positive edge). However, since these connections did not reach significance in the NCT, even when no correction for multiple testing was applied, and due to their weak associations with SI, we refrain from interpreting these edges. Nevertheless, interoceptive awareness remains one of the most central nodes of the network (also indicated by its high bridge EI values (T1: *bridge EI*=0.3, T2: *bridge EI=*0.37; see SM Table S2)), sharing positive edges with feelings of ineffectiveness, depressive as well as anxiety and obsessive-compulsive symptoms (which are in turn strongly linked to depressive symptoms) and thus may influence SI indirectly as well. The tendency observed is that higher scores on the interoceptive awareness scale (indicating poorer interoceptive skills) are associated with increased SI. The association of interoceptive deficits and SI has been demonstrated in both ED and non-ED-populations (Forrest et al., 2015; Ortiz & Smith, 2019), and have even been proposed as a potential predictor of SI (Ortiz & Smith, 2019). This suggests that if patients are struggling with SI, it may be advisable to use interventions strengthening interoceptive skills such as body-focused therapy or mindfulness skills (Fischer et al., 2017; Khalsa et al., 2017; Weng et al., 2020). However, considering the cross-sectional nature of our study, further research is needed to explore this connection and provide more reliable insights.

To conclude, we were able to identify ED-related and general internalizing symptoms as factors associated with SI in AN. However, we did not find relationships between ED-specific symptoms and SI, which is in line with some studies reporting an attenuation or disappearance of the relationship between ED-specific symptoms and suicidality when considering general internalizing symptoms (Bodell et al., 2013; Yao et al., 2016). This further weakens existing literature suggesting ED-specific symptoms to be linked to suicidality (Bühren et al., 2014; Mereu et al., 2022). These studies did not control for other symptoms, which might explain why we were not able to find similar relationships using the network analytic approach. Therefore, when taking ED-related and general internalizing symptoms into account, relationships between ED-specific symptoms and SI might vanish. However, with reference to the IPTS (Joiner, 2005), even though ED-specific and several ED-related symptoms were not directly linked to SI in the current network analysis, ED related factors such as high treatment costs, social isolation or missed school/work due to the illness, might indirectly influence SI via perceived burdensomeness. As these factors were not examined directly, further studies are needed to explore their potential impact on SI in AN.

Evidence indicates that leptin attenuates AN-symptoms (Ehrlich, Burghardt, Schneider, Broecker-Preuss et al., 2009; Fürtjes et al., 2018), and pilot studies suggested that short-term recombinant human leptin treatment may reduce hyperactivity, improve mood and weight-related fears, and support treatment outcomes in AN-patients (Hebebrand et al., 2022; Milos et al., 2020). To evaluate these effects in AN more systematically, a phase-2 randomized, placebo-controlled, quadruple-blind clinical trial using metreleptin is currently underway (trial registration number: NCT06305182). However, our supplementary findings showed no such influence, aligning with another network analysis (Solmi et al., 2018), reporting no significant influence of nutritional status (measured via BMI), and research in mood disorders (Da Graça Cantarelli et al., 2015; Su et al., 2020). As this study measured endogenous leptin, conclusions about exogenous leptin effects remain premature. Further research is needed to clarify the role of both exogenous and endogenous leptin and SI.

### Limitations

The findings presented above should be interpreted in the light of the following limitations. First of all, SI was only assessed via self-report, which could have biased our results due to social desirability effects (i.e., patients concealing their ideation). Measurement error might have been introduced since SI was assessed using three single items taken from two depression questionnaires. However, BDI-II item #9 shows a highly significant correlation with specialized suicidality questionnaires (Desseilles et al., 2012) and the use of SCL-90-R items #15 and #59 to assess SI is well established (Favaro & Santonastaso, 1997; Meng et al., 2013; Milos et al., 2004). Of note, in our approach, the SI node and all others represent subscales (in this case from SCL-90-R and EDI-2 questionnaires) rather than single items, as using numerous individual items would not meet recommended standards for sample size and statistical power in network analyses (Epskamp et al., 2018). Nonetheless, an alternative approach used in some studies (e.g. Smith et al., 2020), where nodes represent individual items, may also be valuable and could yield different results. As we collapsed across multiple facets of SI and, due to multicollinearity concerns, we were unable to examine facet-specific links. Additionally, evidence suggests that the assessment of SI is highly influenced by language, as minor changes in wording can significantly inflate or restrict prevalence estimates (Ammerman et al., 2021). Second, the lack of a uniform definition for ineffectiveness leads to slightly different conceptualizations and different measurements for the construct. Moreover, the AN-sample in this study consisted only of young female patients with - mostly - the restrictive subtype and a severe but short course of disease. Therefore, results might differ in participants of male gender, with binge-eating/purging subtype, with less severe symptoms or with a longer course of illness. Fourth, the inclusion of multiple facets of ED-symptoms and internalizing symptoms, particularly those from the same scale (e.g., subscales of the EDI-2), may result in stronger associations between those related items, potentially inflating the centrality of ED-symptoms and affecting the partial correlations. However, to address this limitation, we ran separate analyses including only ED-related or ED-specific symptom nodes in the networks. The main finding (i.e. ineffectiveness, depressive and anxiety symptoms, but less so ED-specific symptoms being related to SI) was evident in those additional analyses as well. Lastly, our study examined cross-sectional associations at two distinct time points. Given that short-term weight restoration was required for the T2 assessment, selection bias may have been introduced, as individuals with less weight gain might be at a greater risk for SI. Nevertheless, since ideographic network models can support modular, personalized treatments for individuals with SI, future studies should adopt longitudinal within-person network approaches to more precisely identify the key contributing factors (Freichel, 2023; Harris et al., 2025).

## Conclusion

SI plays an important role in individuals suffering from AN, with approximately half of the patients in our sample reporting at least thoughts about killing themselves at the initial assessment and a third struggling with these thoughts even after an intensive treatment period. The results presented above illustrate the interaction of symptoms in AN associated with SI. General internalizing symptoms and ED-related factors influence SI in AN, whereas ED-specific symptoms have less impact. Besides co-occurring depressive and anxiety symptoms, perceived ineffectiveness (ED-related symptom) is a state-independent factor associated with SI in AN. Therefore, it might be important to therapeutically address ineffectiveness with interventions such as shared decision making and empowerment in patients experiencing SI.

## Ethical standards

The authors assert that all procedures contributing to this work comply with the ethical standards of the relevant national and institutional committees on human experimentation and with the Helsinki Declaration of 1975, as revised in 2008.

## Declaration of generative AI and AI-assisted technologies in the writing process

During the preparation of this work the authors used ChatGPT-4o in order to improve the readability and language of the manuscript. After using this tool, the authors reviewed and edited the content as needed and take full responsibility for the content of the published article.

## Declaration of competing interest

The authors declare the following financial interests/personal relationships which may be considered as potential competing interests: Inger Hellerhoff reports financial support was provided by Christina Barz Foundation. Inger Hellerhoff reports financial support was provided by Bayer Foundations. If there are other authors, they declare that they have no known competing financial interests or personal relationships that could have appeared to influence the work reported in this paper.

## Appendix Supplementary materials

Image, application 1

Image, application 2
