# Supplementary material for: The association of eating disorder specific and unspecific symptoms with suicidal ideation in patients with anorexia nervosa
Source: Int J Clin Health Psychol. 2025 Oct 16;25(4):100633. doi: 10.1016/j.ijchp.2025.100633 (PMC12552992; doi:10.1016/j.ijchp.2025.100633)
Supplement: Supplementary file 1 [file mmc1.docx]

**Supplemental Material**

**for the manuscript: “The association of eating disorder specific and unspecific symptoms with suicidal ideation in patients with anorexia nervosa”**

S1

*Methods*

Refeeding intervention

In the admission interview, patients with anorexia nervosa (AN) and their guardians were interviewed regarding the patient’s diet, caloric intake and body weight development in the past weeks. On the basis of this information alongside objective clinical measures (severity of weight loss and current body weight/body mass index (BMI), somatic complications, risk factors for refeeding syndrome), a starting daily caloric intake was determined by an experienced child and adolescent psychiatrist. Higher calorie refeeding as defined by Bargiacchi et al. (2019) (starting daily caloric intake 1500-2400 kcal/day, expected rates of body weight gain 0.5-2 kg/week) was ensured and patients followed a structured meal plan (three meals, three snacks, fluid intake of 2 liters/day), developed in collaboration with a nutritionist, which was individually adjusted in regard to daily caloric intake. Body weight was monitored daily and caloric intake was typically increased every two to three days, depending on individual body weight gain. Hydration status was carefully monitored by regularly examining patients regarding skin turgor, edema, blood pressure and pulse. Furthermore, urine specific gravity was determined daily and electrolytes were determined every other day in the first weeks of inpatient treatment.

While most patients quickly assimilated to the inpatient setting and started eating all served meals on the first or second day of inpatient treatment, a few patients refused to eat individual meals or food items. In this case, patients were encouraged to drink a corresponding quantity of a high-calorie nutrition product (Fresubin Energy Fibre Drink).

In the first weeks of inpatient treatment, the nursing staff portioned and prepared all meals and snacks and supervised food intake. A target BMI in the normal weight range was individually determined, taking BMI development before disease-onset as well as physical markers such as return of menstruation into account. Shortly before reaching their target weight, patients were encouraged to portion and prepare their own meals under the supervision of the nursing staff. After target weight was achieved, patients were encouraged to make their own decisions regarding food items at snack time. In the last weeks of inpatient stay, supervision was progressively reduced to encourage patients to take responsibility for adherence to the meal plan.

Exclusion criteria

Participants were excluded if they had a history of any of the following diagnoses: organic brain syndrome, schizophrenia, substance abuse/dependence, psychosis not otherwise specified, bipolar disorder, bulimia nervosa, or binge-eating disorder. Further exclusion criteria for all participants were an IQ below 85; current substance abuse; inflammatory, neurologic or metabolic illness; chronic medical or neurological illness that could affect appetite, eating behavior or body weight; clinically relevant anemia; pregnancy or breast feeding. Psychoactive medication within four weeks before the study (except for selective serotonin reuptake inhibitors and olanzapine) were additional exclusion criteria for all groups. Additional reasons for drop out from timepoint 1 (T1) to timepoint 2 (T2) included insufficient weight gain (BMI increase of at least 10% was an inclusion criterion) and premature discharge.

S2

*Clinical measures*

The expert form of the Structured Interview for Anorexia and Bulimia Nervosa (SIAB-EX) (Fichter & Quadflieg, 2001a), a well-validated 87-item semi-standardized interview with good inter-rater reliability (range 0.81–0.85) (Fichter & Quadflieg, 2001b), was applied to evaluate the presence and severity of current (within three months before the study) eating-related psychopathology (including assessment of AN-subtype and physical activity used for descriptive statistics and multiple imputation of left-censored leptin values). SIAB-EX interviews were conducted by clinically experienced and trained research assistants under supervision of a child and adolescent psychiatrist. Co-existing psychiatric diagnoses other than eating disorders were derived according to standard practice from medical records and confirmed by a board-licensed child and adolescent psychiatrist with over 10 years of clinical experience.

Socio-economic status (SES) was computed as the mean of two indicators: the average of highest educational level and occupational skill level. Both were rated on a scale from 0 to 5, with higher values indicating higher educational attainment and more complex or skilled occupations. As many participants were adolescents still in education and living with their parents or guardians, parental SES was used as a proxy, drawing on the framework outlined by Ganzeboom et al. (1992).

Plasma leptin level was measured in fasting venous blood samples collected into EDTA vacutainer tubes between 7–9 a.m. and within 96h after treatment initiation, using a commercially available enzyme-linked immunosorbent assay (ELISA, BioVendor Research and Diagnostic Products, Brno/Czech Republic) with intra-/inter-assay variation coefficients <6%. It was processed as follows: addition of the serine protease inhibitor aprotinin, centrifugation (at ϑ=5°C and a=2,500*g for 15min), aliquotation into Eppendorf Tubes, storage at ϑ=-80°C until laboratory analysis. Plasma samples were processed in four batches at the same laboratory (Magdeburg/Germany 2014, 2015, 2017, and 2021; analyses accounted for leptin batch effects [covariance approach] to exclude potential confounding). Plasma leptin levels were measured (as single measurement) using the same enzyme-linked immunosorbent assay (BioVendor Research and Diagnostic Products, Brno/Czech Republic) with intra-/inter-assay variation coefficients <6% and a lower limit of detection (LOD) of 0.20μg/L across all batches. Non-detectable leptin concentrations below the lower limit of detection of the leptin assay (LOD=0.20 μg/L) occurred in 61 of 379 leptin-samples (16.1%) and were imputed using a quantile regression multiple imputation approach for left-censored missing data (QRILC). QRILC performs random draws from a truncated distribution with parameters estimated using quantile regression (derived from the distribution of existing leptin concentrations within detection range, please note that no further covariates were introduced in the imputation model). QRILC was conducted in R with the help of package "imputeLCMD" (Lazar, 2015). A Gibbs sampler based approach (Wei et al., 2018) with n=100 iterative draws per value from the specified truncated distribution was then used to update the initialized values from QRILC and to ensure that the imputed leptin values were positive (on the original scale, i.e., >0) and below LOD. Randomly missing/ unavailable leptin values due to unavailable blood samples were not imputed (*n_T1_=*84, *n_T2_*=67). For analyses, leptin values were logarithmically transformed; log10-leptin was normally distributed.

S3

*Psychometric properties of SI score*

While the original instruments from which the items were drawn (SCL-90-R and BDI-II) were not designed exclusively for suicidal ideation (SI) assessment, they are well-established indicators of SI (Bertoli et al., 2016; Favaro & Santonastaso, 1997; Kämpfer et al., 2016; Meng et al., 2013; Milos et al., 2004; Miotto et al., 2003). Notably, BDI-II item #9 shows a strong correlation with dedicated suicidality scales (correlation with Beck’s scale for SI (total score): r=0.48, p=0.0001) (Desseilles et al., 2012), supporting its relevance.

The SI construct in our study was operationalized using three ordinal indicators — two items from the SCL-90-R (scl90r_question_15, scl90r_question_59) and one from the BDI-II (bdi2_question_9) — which were z-transformed. Given the high conceptual overlap and intercorrelations among the three selected items, we combined them into a single composite SI node rather than including them separately, which would introduce redundancy and violate independence assumptions. Visual inspection and exploratory factor analysis supported a unidimensional latent structure, with strong loadings for all items (scl90r_question_15: 0.91, scl90r_question_59: 0.90, bdi2_question_9: 0.73). Internal consistency was assessed with McDonald’s omega (ω = .88 (95% CI [.86, .91])), confirming reliable measurement of the SI construct (Dunn et al., 2013; McDonald, 2013). Additionally, convergent validity was supported by a significant positive correlation between the SI score and the total BDI score (excluding BDI item #9) (Ρ = 0.62, p < 0.001), demonstrating that the SI score captures clinically meaningful SI related to overall depressive symptoms. Divergent validity was evidenced by a weak, non-significant correlation between the SI score and BMI-SDS (Ρ = -0.03, p = 0.60), supporting the specificity of the SI score for SI rather than illness severity in our AN-patients.

Together, these findings provide robust psychometric support for the SI score, justifying its use as a single, reliable, and valid construct in our network analysis and enhancing the interpretability of SI within the broader symptom network.

S4

*Suicidal ideation (SI) network – eating symptomatology; depressive, anxiety, and obsessive-compulsive-symptoms*

Other edges that were significantly different between the two timepoints were bulimia – ineffectiveness (*P*=0.003), bulimia – perfectionism (*P*=0.03), perfectionism – maturity fears (*P*=0.05) and body dissatisfaction – depression (*P*=0.02).

S5

*Supplementary analysis: adding leptin to the network*

Networks are depicted in SM Figures S3 and S4. The nodes with the highest expected influence in both networks were the same as in the original network (see SM Figure S5), i.e. ineffectiveness (T1: M=1.31, T2: M=1.22), depressive symptoms (T1: M=1.3, T2: M=1.39), and interoception (T1: M=1.15, T2: M=1.15). Of particular interest to our hypothesis was the weak positive edge of leptin and SI at T1 and T2. However, when comparing this effect to other nodes in the network, the connection was of negligible magnitude. For more detail see SM (Tables S6-S8, Figures S3-S12).

When comparing both leptin networks, no significant differences were found in global strength and network invariance (S=0.44, *P*=0.35; M=0.18, *P*=0.48; see SM Table S8 for further details). Without p-adjust methods, a significant difference regarding the leptin – bulimia edge was found *(P*=0.04, stronger positive edge at T2). Moreover, significant differences were observed in the edges SI – OC symptoms (*P*=0.02; with no edge at T1 and a positive edge at T2) and SI – depressive symptoms (*P*=0.03, with a positive edge at T1 and a stronger positive edge at T2).

Based on the findings above, four exploratory analyses were conducted to delve deeper into the relationship between leptin and SI while also considering potential effects of BMI. We formed three networks for this purpose: in network 1, both leptin and BMI-SDS were included in the SI network (SM Figures S6, S7); in network 2, leptin was adjusted for BMI-SDS (SM Figures S8-S9); and in network 3, leptin was excluded while BMI-SDS was included in the network (SM Figures S10-S11). When computing the network analysis while including BMI and leptin, the weak connection between leptin and SI was still evident at T1 and T2. This connection persisted when residualizing BMI out of leptin. When excluding leptin and including only BMI into the network analysis, no edge between BMI and SI was evident. A scatter plot illustrating the relationship between leptin and SI was generated, revealing no outliers that could account for the obtained results (SM Figure S12).

**Tables**

Table S1

*Mean and standard deviation for each variable*

| Variable | T1 | T2 | p-value | r-value |
| --- | --- | --- | --- | --- |
| Age, y | 15.89±2.29 | 16.15±2.28 |  |  |
| BMI, kg/m^2^ | 14.66±1.32 | 18.84±1.37 | <2.2e^-16^ | 0.61 |
| BMI-SDS | -3.16±1.08 | -0.73±0.69 | <2.2e^-16^ | 0.61 |
| Leptin, µg/L | 1.66±2.36 | 12.36±7.25 | <2.2e^-16^ | 0.56 |
| EDI-2 total | 212.77±43.98 | 202.21±47.92 | 2.618e^-05^ | 0.21 |
| SI | 0.44±0.56 | 0.41±0.6 | 0.48 |  |
| SCL-90-R DEP | 2.6±0.86 | 2.03±0.81 | <2.2e^-16^ | 0.44 |
| SCL-90-R ANX | 0.91±0.75 | 0.74±0.74 | 0.001038 | 0.16 |
| SCL-90-R OCS | 1.16±0.82 | 0.78±0.66 | 4.489e^-13^ | 0.36 |
| BDI-2 total | 23.93±11.13 | 16.56±12.3 | <2.2e^-16^ | 0.43 |
| *ED-specific EDI_subscales* | | | | |
| EDI2_DT | 29.4±9.47 | 27.01±10.1 | 6.314e^-05^ | 0.20 |
| EDI2_B | 11.29±5.29 | 9.53±3.01 | 3.734e^-07^ | 0.26 |
| EDI2_BD | 37.55±10.34 | 38.41±11.67 | 0.26 |  |
| *ED-related EDI_subscales* | | | | |
| EDI2_INF | 32.8±9.98 | 31.44±10.58 | 0.002846 | 0.15 |
| EDI2_P | 19.42±5.33 | 18.79±5.34 | 0.02877 | 0.11 |
| EDI2_IPD | 21.85±6.67 | 21.98±6.84 | 0.72 |  |
| EDI2_ICP | 32.27±9.58 | 28.01±9.49 | 1.186e^-13^ | 0.37 |
| EDI2_MF | 28.11±6.28 | 27±6.24 | 0.001247 | 0.16 |

*Note.* Mean values±standard deviation for each variable are shown separately for each timepoint. Differences were tested using Wilcoxon signed-rank test.

*Abbreviations*. T1, timepoint 1 (admission); T2, timepoint 2 (body mass index increase of at least 10%); p-value, significance of difference; r-value, effect size of observed effect; BMI, body mass index; BMI-SDS, body mass index standard deviation score; SI, suicidal ideation as measured via BDI-II (Beck Depression Inventory-II) item #9; SCL-90-R DEP, depressive symptom score of SCL-90-R (generated without item #15); SCL-90-R ANX, anxiety symptom score of SCL-90-R; SCL-90-R OCS, obsessive-compulsive symptom score of SCL-90-R; EDI-2 DT, EDI-2 scale drive for thinness; EDI-2 B, EDI-2 scale bulimia; EDI-2 BD, EDI-2 scale body dissatisfaction; EDI-2 INF, EDI-2 scale ineffectiveness; EDI-2 P, EDI-2 scale perfectionism; EDI-2 IPD, EDI-2 scale interpersonal distrust; EDI-2 ICP, EDI-2 scale interoception; EDI-2 MF, EDI-2 scale maturity fears.

Table S2

Bridge expected influence of nodes at timepoint 1 and timepoint 2

| Nodes | Bridge expected influence at T1 | Bridge expected influence at T2 |
| --- | --- | --- |
| SI | 0.51 | 0.52 |
| DT | 0.06 | 0.08 |
| B | 0.06 | -0.08 |
| BD | 0.01 | -0.04 |
| INF | 0.42 | 0.44 |
| P | 0.21 | 0.08 |
| IPD | 0.04 | 0.00 |
| ICP | 0.30 | 0.37 |
| MF | 0.00 | 0.06 |
| OCS | 0.37 | 0.28 |
| ANX | 0.15 | 0.14 |
| DEP | 0.63 | 0.70 |

*Abbreviations*. SI, suicidal ideation score as measured via BDI-II item #9 and SCL90-R items #15 und #59; DT, EDI-2 scale drive for thinness; B, EDI-2 scale bulimia; BD, EDI-2 scale body dissatisfaction; INF, EDI-2 scale ineffectiveness; P, EDI-2 scale perfectionism; IPD, EDI-2 scale interpersonal distrust; ICP, EDI-2 scale interoception; MF, EDI-2 scale maturity fears; DEP, depressive symptom score of SCL-90-R (generated without item #15); ANX, anxiety symptom score of SCL-90-R; OCS, obsessive-compulsive symptom score of SCL-90-R.

Table S3

*Partial estimates original network T1*

|  | SI | DT | B | BD | INF | P | IPD | ICP | MF | OCS | ANX | DEP |
| --- | --- | --- | --- | --- | --- | --- | --- | --- | --- | --- | --- | --- |
| SI | 0 | 0 | 0 | 0 | 0.19 | 0.04 | 0 | 0 | 0 | 0 | 0.12 | 0.16 |
| DT | 0 | 0 | 0 | 0.58 | 0.02 | 0 | 0 | 0.14 | 0 | 0.02 | 0 | 0.04 |
| B | 0 | 0 | 0 | 0.03 | 0 | 0 | 0 | 0.18 | 0 | 0.06 | 0 | 0 |
| BD | 0 | 0.58 | 0.03 | 0 | 0.17 | 0 | 0 | 0 | 0 | 0 | 0 | 0.01 |
| INF | 0.19 | 0.02 | 0 | 0.17 | 0 | 0.04 | 0.29 | 0.21 | 0.21 | 0 | 0 | 0.23 |
| P | 0.04 | 0 | 0 | 0 | 0.04 | 0 | 0.01 | 0.12 | 0 | 0.09 | 0 | 0.08 |
| IPD | 0 | 0 | 0 | 0 | 0.29 | 0.01 | 0 | 0.22 | 0 | 0.01 | 0.03 | 0 |
| ICP | 0 | 0.14 | 0.18 | 0 | 0.21 | 0.12 | 0.22 | 0 | 0 | 0.19 | 0 | 0.11 |
| MF | 0 | 0 | 0 | 0 | 0.21 | 0 | 0 | 0 | 0 | 0 | 0 | 0 |
| OCS | 0 | 0.02 | 0.06 | 0 | 0 | 0.09 | 0.01 | 0.19 | 0 | 0 | 0.34 | 0.37 |
| ANX | 0.12 | 0 | 0 | 0 | 0 | 0 | 0.03 | 0 | 0 | 0.34 | 0 | 0.28 |
| DEP | 0.16 | 0.04 | 0 | 0.01 | 0.23 | 0.08 | 0 | 0.11 | 0 | 0.37 | 0.28 | 0 |

*Note.* Partial estimates for each edge at timepoint 1 (admission).

*Abbreviations*. SI, suicidal ideation score as measured via BDI-II item #9 and SCL90-R items #15 und #59; DT, EDI-2 scale drive for thinness; B, EDI-2 scale bulimia; BD, EDI-2 scale body dissatisfaction; INF, EDI-2 scale ineffectiveness; P, EDI-2 scale perfectionism; IPD, EDI-2 scale interpersonal distrust; ICP, EDI-2 scale interoception; MF, EDI-2 scale maturity fears; DEP, depressive symptom score of SCL-90-R (generated without item #15); ANX, anxiety symptom score of SCL-90-R; OCS, obsessive-compulsive symptom score of SCL-90-R.

Table S4

*Partial estimates original network T2*

|  | SI | DT | B | BD | INF | P | IPD | ICP | MF | OCS | ANX | DEP |
| --- | --- | --- | --- | --- | --- | --- | --- | --- | --- | --- | --- | --- |
| SI | 0 | 0 | -0.07 | -0.03 | 0.21 | -0.04 | 0 | 0.08 | 0 | 0 | 0.11 | 0.27 |
| DT | 0 | 0 | 0 | 0.66 | 0 | 0.06 | 0 | 0.1 | 0.05 | 0 | 0 | 0.08 |
| B | -0.07 | 0 | 0 | 0.03 | 0.09 | 0.13 | 0 | 0.18 | 0 | 0.06 | -0.07 | 0 |
| BD | -0.03 | 0.66 | 0.03 | 0 | 0.24 | 0 | 0 | 0 | 0.05 | 0 | -0.02 | 0 |
| INF | 0.21 | 0 | 0.09 | 0.24 | 0 | 0.02 | 0.26 | 0.23 | 0.07 | 0 | 0 | 0.23 |
| P | -0.04 | 0.06 | 0.13 | 0 | 0.02 | 0 | 0 | 0.11 | 0.14 | 0 | 0.06 | 0.06 |
| IPD | 0 | 0 | 0 | 0 | 0.26 | 0 | 0 | 0.22 | -0.09 | 0 | 0 | 0 |
| ICP | 0.08 | 0.1 | 0.18 | 0 | 0.23 | 0.11 | 0.22 | 0 | 0 | 0.22 | 0.07 | 0 |
| MF | 0 | 0.05 | 0 | 0.05 | 0.07 | 0.14 | -0.09 | 0 | 0 | 0 | 0 | 0.06 |
| OCS | 0 | 0 | 0.06 | 0 | 0 | 0 | 0 | 0.22 | 0 | 0 | 0.35 | 0.38 |
| ANX | 0.11 | 0 | -0.07 | -0.02 | 0 | 0.06 | 0 | 0.07 | 0 | 0.35 | 0 | 0.29 |
| DEP | 0.27 | 0.08 | 0 | 0 | 0.23 | 0.06 | 0 | 0 | 0.06 | 0.38 | 0.29 | 0 |

*Note*. Partial estimates for each edge at timepoint 2 (body mass index increase of at least 10%).
*Abbreviations*. SI, suicidal ideation score as measured via BDI-II item #9 and SCL90-R items #15 und #59; DT, EDI-2 scale drive for thinness; B, EDI-2 scale bulimia; BD, EDI-2 scale body dissatisfaction; INF, EDI-2 scale ineffectiveness; P, EDI-2 scale perfectionism; IPD, EDI-2 scale interpersonal distrust; ICP, EDI-2 scale interoception; MF, EDI-2 scale maturity fears; DEP, depressive symptom score of SCL-90-R (generated without item #15); ANX, anxiety symptom score of SCL-90-R; OCS, obsessive-compulsive symptom score of SCL-90-R.

Table S5

*Network comparison tests original network*

|  | Network invariance test | | Global strength invariance test | | | |
| --- | --- | --- | --- | --- | --- | --- |
|  | M | p-value | Global strength group1 | Global strength group2 | S | p-value |
| With p-adjust | 0.14 | 0.60 | 4.6 | 5.47 | 0.87 | 0.05 |
| Without p-adjust | 0.14 | 0.61 | 4.6 | 5.47 | 0.87 | 0.04 |

Table S6

*Partial estimates original network with leptin T1*

|  | SI | LEP | DT | B | BD | INF | P | IPD | ICP | MF | OCS | ANX | DEP |
| --- | --- | --- | --- | --- | --- | --- | --- | --- | --- | --- | --- | --- | --- |
| SI | 0 | 0.01 | 0 | 0 | 0 | 0.2 | 0.05 | 0.02 | 0 | 0 | 0 | 0.11 | 0.17 |
| LEP | 0.01 | 0 | 0 | 0.02 | 0 | 0 | 0 | 0 | 0 | 0 | 0 | 0 | 0 |
| DT | 0 | 0 | 0 | 0.02 | 0.55 | 0.05 | 0 | 0 | 0.15 | 0 | 0.03 | 0 | 0.03 |
| B | 0 | 0.02 | 0.02 | 0 | 0.04 | 0 | 0 | 0 | 0.2 | 0 | 0.1 | 0 | 0 |
| BD | 0 | 0 | 0.55 | 0.04 | 0 | 0.15 | 0 | 0 | 0 | 0 | 0 | 0 | 0.02 |
| INF | 0.2 | 0 | 0.05 | 0 | 0.15 | 0 | 0 | 0.24 | 0.21 | 0.2 | 0 | 0 | 0.26 |
| P | 0.05 | 0 | 0 | 0 | 0 | 0 | 0 | 0.05 | 0.05 | 0.03 | 0.14 | 0.06 | 0.08 |
| IPD | 0.02 | 0 | 0 | 0 | 0 | 0.24 | 0.05 | 0 | 0.23 | 0 | 0.01 | 0 | 0 |
| ICP | 0 | 0 | 0.15 | 0.2 | 0 | 0.21 | 0.05 | 0.23 | 0 | 0 | 0.19 | 0.03 | 0.11 |
| MF | 0 | 0 | 0 | 0 | 0 | 0.2 | 0.03 | 0 | 0 | 0 | 0 | 0 | 0 |
| OCS | 0 | 0 | 0.03 | 0.1 | 0 | 0 | 0.14 | 0.01 | 0.19 | 0 | 0 | 0.31 | 0.36 |
| ANX | 0.11 | 0 | 0 | 0 | 0 | 0 | 0.06 | 0 | 0.03 | 0 | 0.31 | 0 | 0.27 |
| DEP | 0.17 | 0 | 0.03 | 0 | 0.02 | 0.26 | 0.08 | 0 | 0.11 | 0 | 0.36 | 0.27 | 0 |

*Note.* Partial estimates for each edge at timepoint 1 (admission).

*Abbreviations*. SI, suicidal ideation score as measured via BDI-II item #9 and SCL90-R items #15 und #59; LEP, endogenous leptin; DT, EDI-2 scale drive for thinness; B, EDI-2 scale bulimia; BD, EDI-2 scale body dissatisfaction; INF, EDI-2 scale ineffectiveness; P, EDI-2 scale perfectionism; IPD, EDI-2 scale interpersonal distrust; ICP, EDI-2 scale interoception; MF, EDI-2 scale maturity fears; DEP, depressive symptom score of SCL-90-R (generated without item #15); ANX, anxiety symptom score of SCL-90-R; OCS, obsessive-compulsive symptom score of SCL-90-R.

Table S7

*Partial estimates original network with leptin T2*

|  | SI | LEP | DT | B | BD | INF | P | IPD | ICP | MF | OCS | ANX | DEP |
| --- | --- | --- | --- | --- | --- | --- | --- | --- | --- | --- | --- | --- | --- |
| SI | 0 | 0.02 | 0.01 | -0.05 | 0 | 0.14 | 0 | 0 | 0.03 | 0 | 0.06 | 0.05 | 0.33 |
| LEP | 0.02 | 0 | 0 | 0.1 | 0 | 0 | 0 | 0 | 0 | 0 | 0 | 0 | 0 |
| DT | 0.01 | 0 | 0 | 0 | 0.66 | 0 | 0.05 | 0 | 0.14 | 0.05 | 0 | 0.01 | 0.06 |
| B | -0.05 | 0.1 | 0 | 0 | 0.02 | 0.11 | 0.12 | 0 | 0.23 | 0 | 0 | 0 | 0 |
| BD | 0 | 0 | 0.66 | 0.02 | 0 | 0.21 | 0 | 0 | 0 | 0.05 | 0 | 0 | 0 |
| INF | 0.14 | 0 | 0 | 0.11 | 0.21 | 0 | 0.11 | 0.24 | 0.18 | 0.02 | 0 | 0 | 0.22 |
| P | 0 | 0 | 0.05 | 0.12 | 0 | 0.11 | 0 | 0 | 0.05 | 0.15 | 0.04 | 0.06 | 0.04 |
| IPD | 0 | 0 | 0 | 0 | 0 | 0.24 | 0 | 0 | 0.23 | 0 | 0.02 | 0 | 0 |
| ICP | 0.03 | 0 | 0.14 | 0.23 | 0 | 0.18 | 0.05 | 0.23 | 0 | 0 | 0.26 | 0.02 | 0.01 |
| MF | 0 | 0 | 0.05 | 0 | 0.05 | 0.02 | 0.15 | 0 | 0 | 0 | 0 | 0 | 0.04 |
| OCS | 0.06 | 0 | 0 | 0 | 0 | 0 | 0.04 | 0.02 | 0.26 | 0 | 0 | 0.28 | 0.32 |
| ANX | 0.05 | 0 | 0.01 | 0 | 0 | 0 | 0.06 | 0 | 0.02 | 0 | 0.28 | 0 | 0.37 |
| DEP | 0.33 | 0 | 0.06 | 0 | 0 | 0.22 | 0.04 | 0 | 0.01 | 0.04 | 0.32 | 0.37 | 0 |

*Note*. Partial estimates for each edge at timepoint 2 (body mass index increase of at least 10%).

*Abbreviations*. SI, suicidal ideation score as measured via BDI-II item #9 and SCL90-R items #15 und #59; LEP, endogenous leptin; DT, EDI-2 scale drive for thinness; B, EDI-2 scale bulimia; BD, EDI-2 scale body dissatisfaction; INF, EDI-2 scale ineffectiveness; P, EDI-2 scale perfectionism; IPD, EDI-2 scale interpersonal distrust; ICP, EDI-2 scale interoception; MF, EDI-2 scale maturity fears; DEP, depressive symptom score of SCL-90-R (generated without item #15); ANX, anxiety symptom score of SCL-90-R; OCS, obsessive-compulsive symptom score of SCL-90-R.

Table S8

*Network comparison tests original network with leptin*

|  | Network invariance test | | Global strength invariance test | | | |
| --- | --- | --- | --- | --- | --- | --- |
|  | M | p-value | Global strength group1 | Global strength group2 | S | p-value |
| With p-adjust | 0.18 | 0.47 | 4.71 | 5.16 | 0.44 | 0.34 |
| Without p-adjust | 0.18 | 0.48 | 4.71 | 5.16 | 0.44 | 0.36 |

**Figures**

*
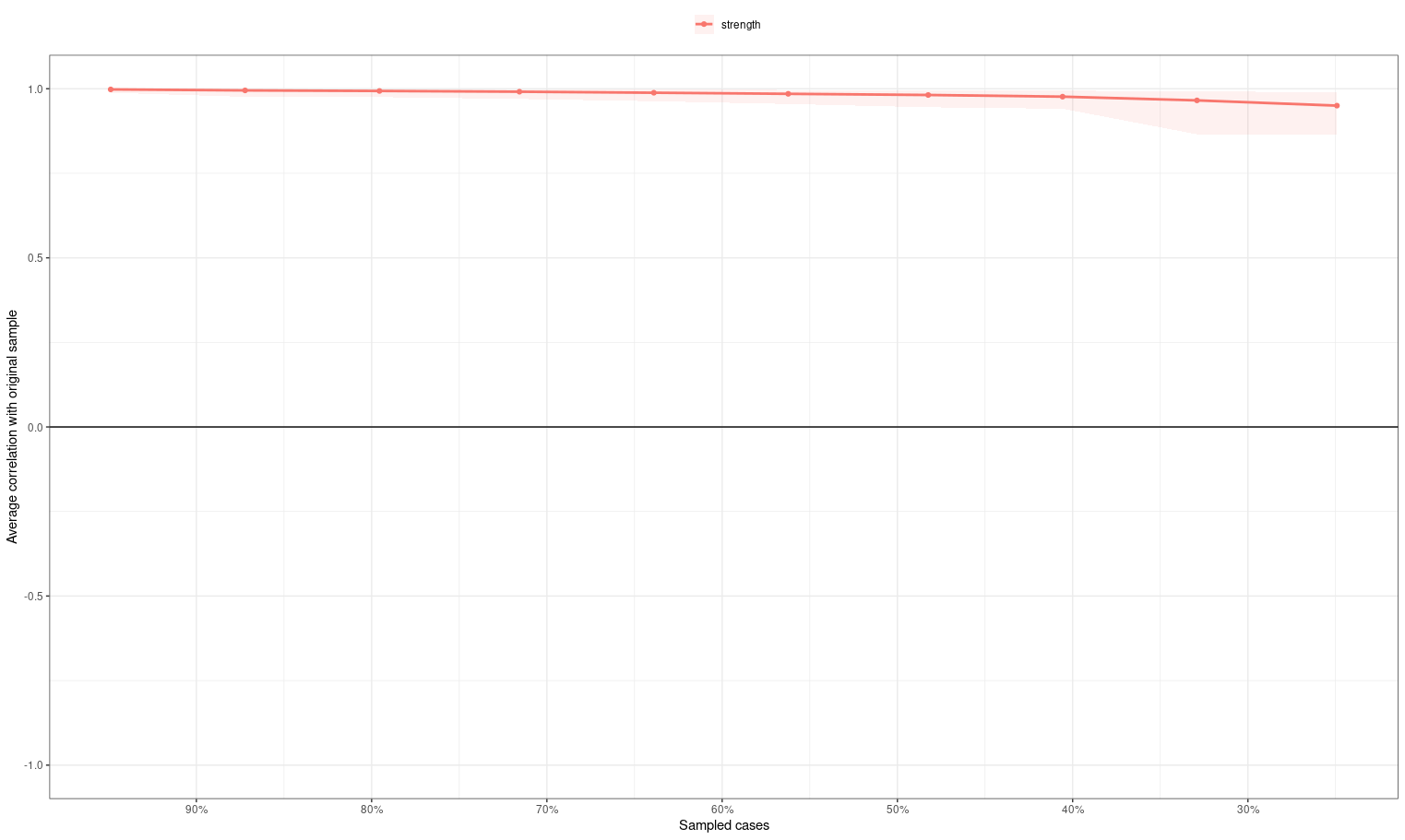
*

Figure S1

Edge stability network at timepoint 1 (main model)

*
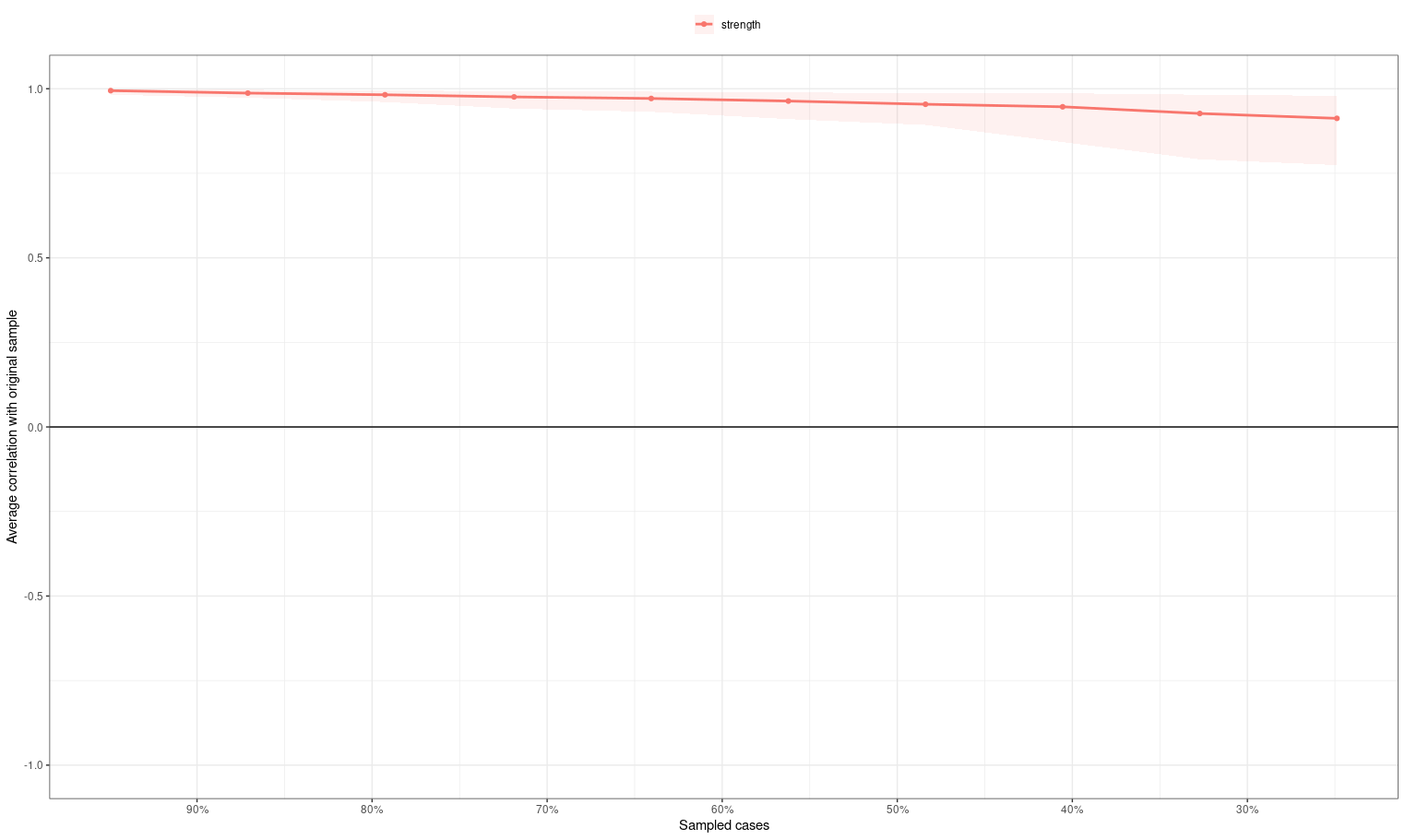
*

Figure S2

Edge stability network at timepoint 2 (main model)

*
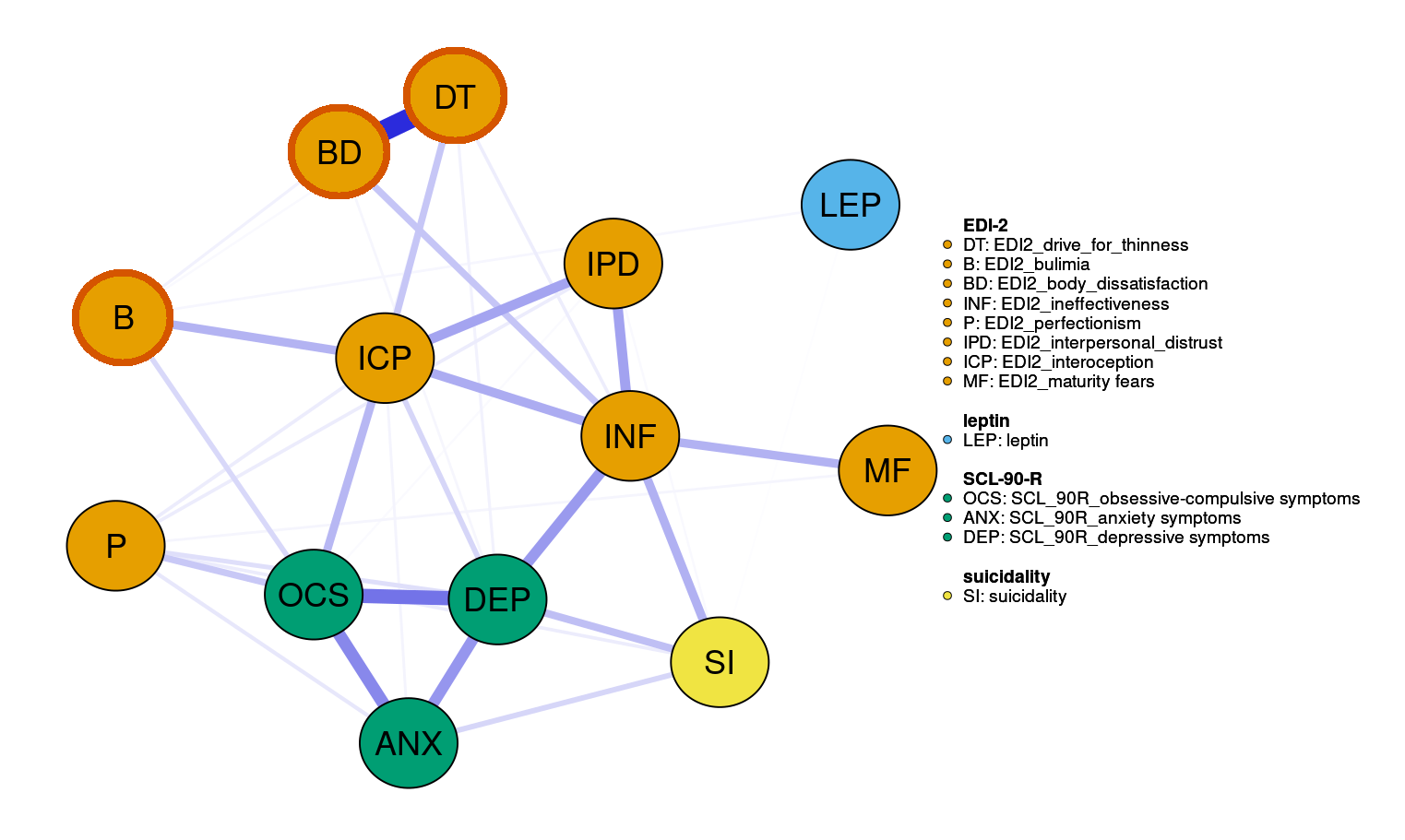
*

Figure S3

Estimated network including leptin of total patient population (n=229) at timepoint 1 (admission). Green nodes: Subscales of Symptom Checklist-90-Revised. Orange nodes: Subscales of Eating Disorder Inventory-2 (EDI-2). Darker frame around orange nodes: ED-specific symptoms (EDI-2). Blue lines: positive partial associations. Red lines: negative partial associations. Thickness and strength of lines: strength of the partial correlation. Correlation Stability coefficient (CS-coefficient) strength: 0.75.

*Note.* LEP, leptin; SI, suicidal ideation; DEP, depressive symptom score of SCL-90-R (generated without item #15); ANX, anxiety symptom score of SCL-90-R; OCS, obsessive-compulsive symptom score of SCL-90-R; DT, EDI-2 scale drive for thinness; B, EDI-2 scale bulimia; BD, EDI-2 scale body dissatisfaction; INF, EDI-2 scale ineffectiveness; P, EDI-2 scale perfectionism; IPD, EDI-2 scale interpersonal distrust; ICP, EDI-2 scale interoception; MF, EDI-2 scale maturity fears.

*
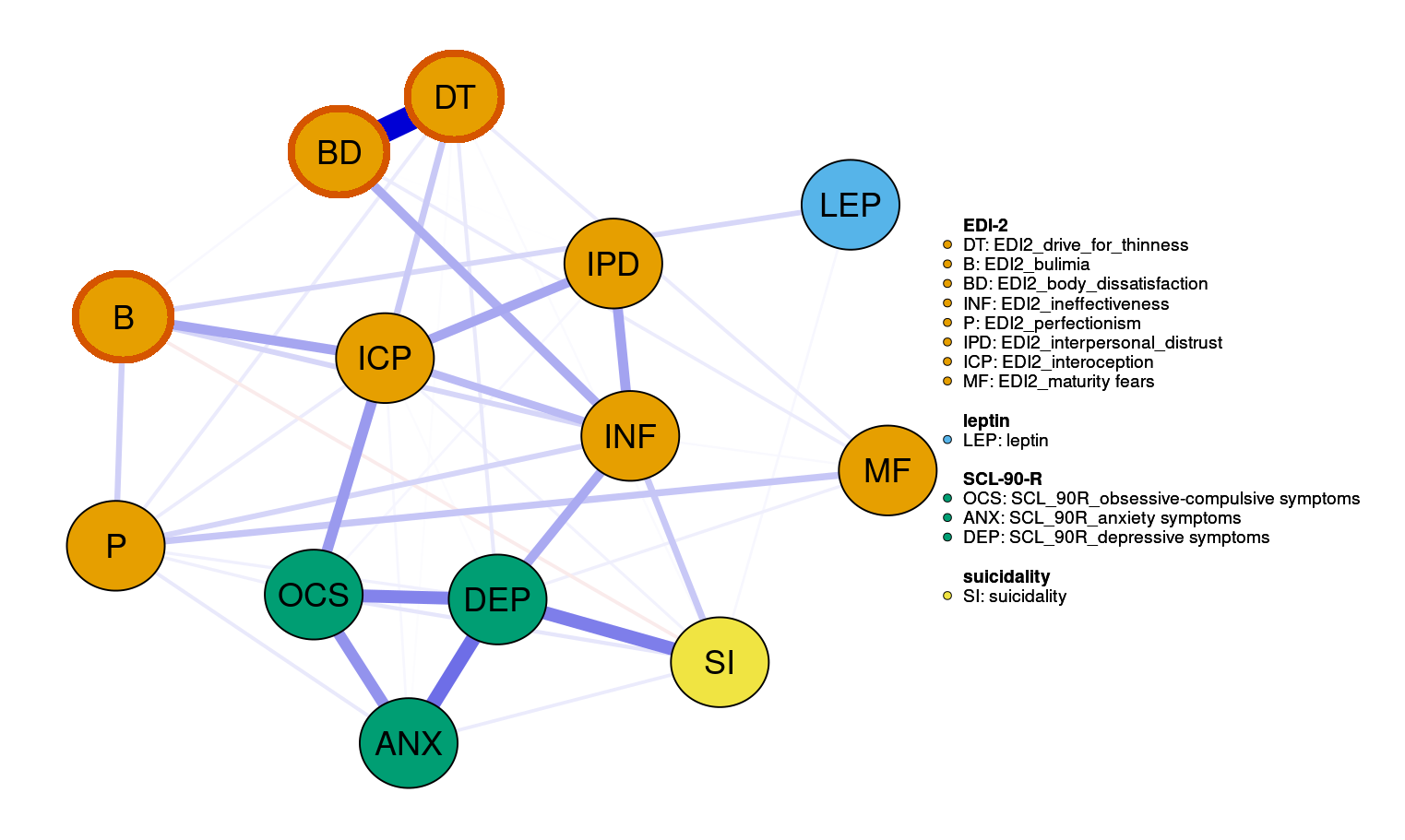
*

Figure S4

Estimated network including leptin of total patient population (n=150) at timepoint 2. Green nodes: Subscales of Symptom Checklist-90-Revised. Orange nodes: Subscales of Eating Disorder Inventory-2 (EDI-2). Darker frame around orange nodes: ED-specific symptoms (EDI-2). Blue lines: positive partial associations. Red lines: negative partial associations. Thickness and strength of lines: strength of the partial correlation. CS-coefficient strength: 0.67.

*Note.* LEP, leptin; SI, suicidal ideation; DEP, depressive symptom score of SCL-90-R (generated without item #15); ANX, anxiety symptom score of SCL-90-R; OCS, obsessive-compulsive symptom score of SCL-90-R; DT, EDI-2 scale drive for thinness; B, EDI-2 scale bulimia; BD, EDI-2 scale body dissatisfaction; INF, EDI-2 scale ineffectiveness; P, EDI-2 scale perfectionism; IPD, EDI-2 scale interpersonal distrust; ICP, EDI-2 scale interoception; MF, EDI-2 scale maturity fears.


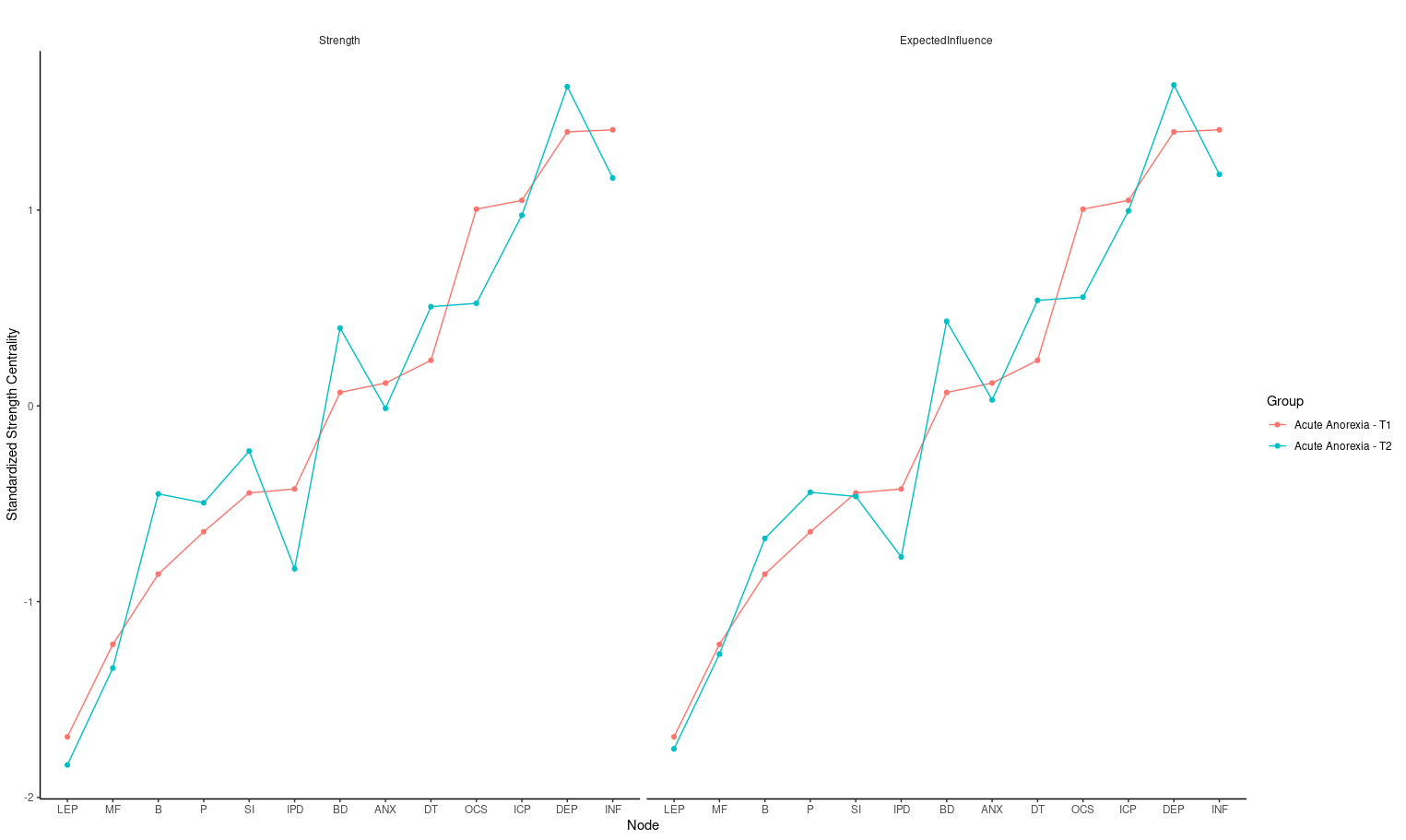


Figure S5

Plotted centrality indices of each node in suicidal ideation networks at timepoint 1 and timepoint 2 – including leptin. Red line: acute anorexia nervosa patients at timepoint 1; blue line: anorexia nervosa patients at timepoint 2.

*Note.* LEP, leptin; MF, EDI-2 scale maturity fears; B, EDI-2 scale bulimia; P, EDI-2 scale perfectionism; SI, suicidal ideation; IPD, EDI-2 scale interpersonal distrust; BD, EDI-2 scale body dissatisfaction; ANX, anxiety symptom score of SCL-90-R ; DT, EDI-2 scale drive for thinness; OCS, obsessive-compulsive symptom score of SCL-90-R ; ICP, EDI-2 scale interoceptive awareness; DEP, depressive symptom score of SCL-90-R (generated without item #15); INF, EDI-2 scale ineffectiveness.

*
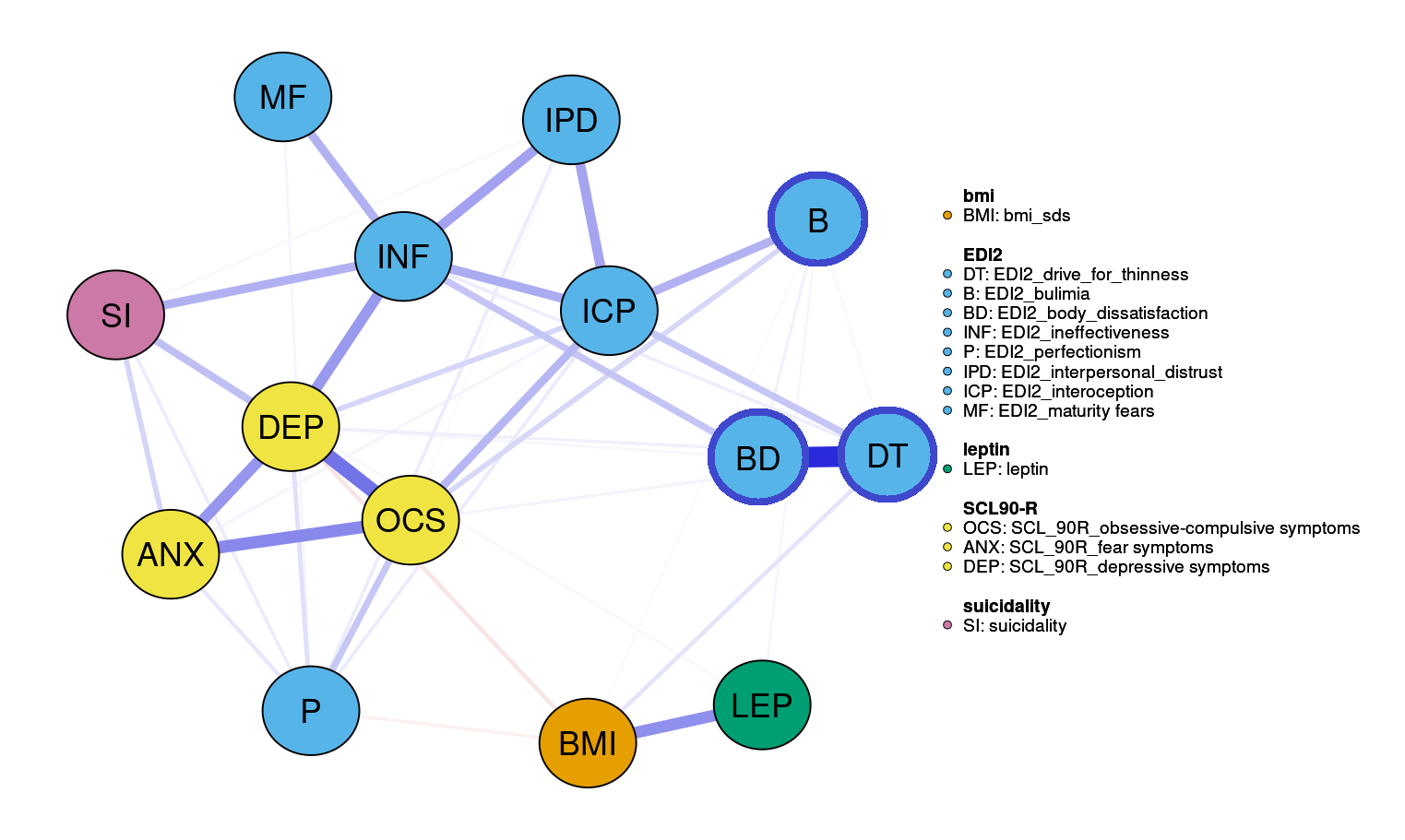
*

Figure S6

Estimated network of total patient population (n=229) at timepoint 1 including leptin and BMI. Yellow nodes: Subscales of Symptom Checklist-90-Revised. Blue nodes: Subscales of Eating Disorder Inventory-2 (EDI-2). Darker frame around blue nodes: ED-specific symptoms (EDI-2). Blue lines: positive partial associations. Red lines: negative partial associations. Thickness and strength of lines: strength of the partial correlation. CS-coefficient strength: 0.75.

*Note.* SI, suicidal ideation; DEP, depressive symptom score of SCL-90-R (generated without item #15); ANX, anxiety symptom score of SCL-90-R; OCS, obsessive-compulsive symptom score of SCL-90-R; DT, EDI-2 scale drive for thinness; B, EDI-2 scale bulimia; BD, EDI-2 scale body dissatisfaction; INF, EDI-2 scale ineffectiveness; P, EDI-2 scale perfectionism; IPD, EDI-2 scale interpersonal distrust; ICP, EDI-2 scale interoception; MF, EDI-2 scale maturity fears; LEP, leptin; BMI, body mass index.

*
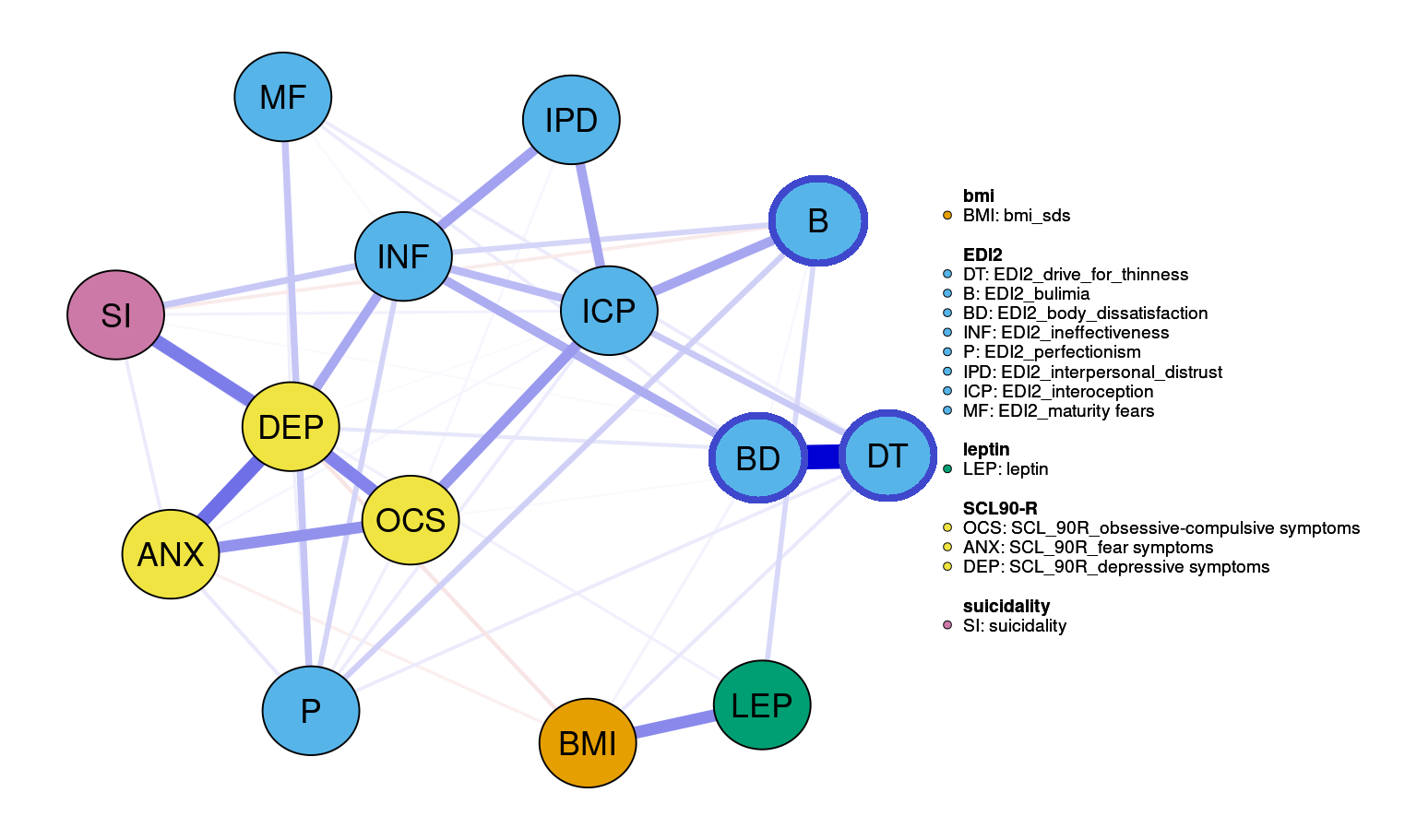
*

Figure S7

Estimated network of total patient population (n=150) at timepoint 2 including leptin and BMI. Yellow nodes: Subscales of Symptom Checklist-90-Revised. Blue nodes: Subscales of Eating Disorder Inventory-2 (EDI-2). Darker frame around blue nodes: ED-specific symptoms (EDI-2). Blue lines: positive partial associations. Red lines: negative partial associations. Thickness and strength of lines: strength of the partial correlation. CS-coefficient strength: 0.67.

*Note.* SI, suicidal ideation; DEP, depressive symptom score of SCL-90-R (generated without item #15); ANX, anxiety symptom score of SCL-90-R; OCS, obsessive-compulsive symptom score of SCL-90-R; DT, EDI-2 scale drive for thinness; B, EDI-2 scale bulimia; BD, EDI-2 scale body dissatisfaction; INF, EDI-2 scale ineffectiveness; P, EDI-2 scale perfectionism; IPD, EDI-2 scale interpersonal distrust; ICP, EDI-2 scale interoception; MF, EDI-2 scale maturity fears; LEP, leptin; BMI, body mass index.

*
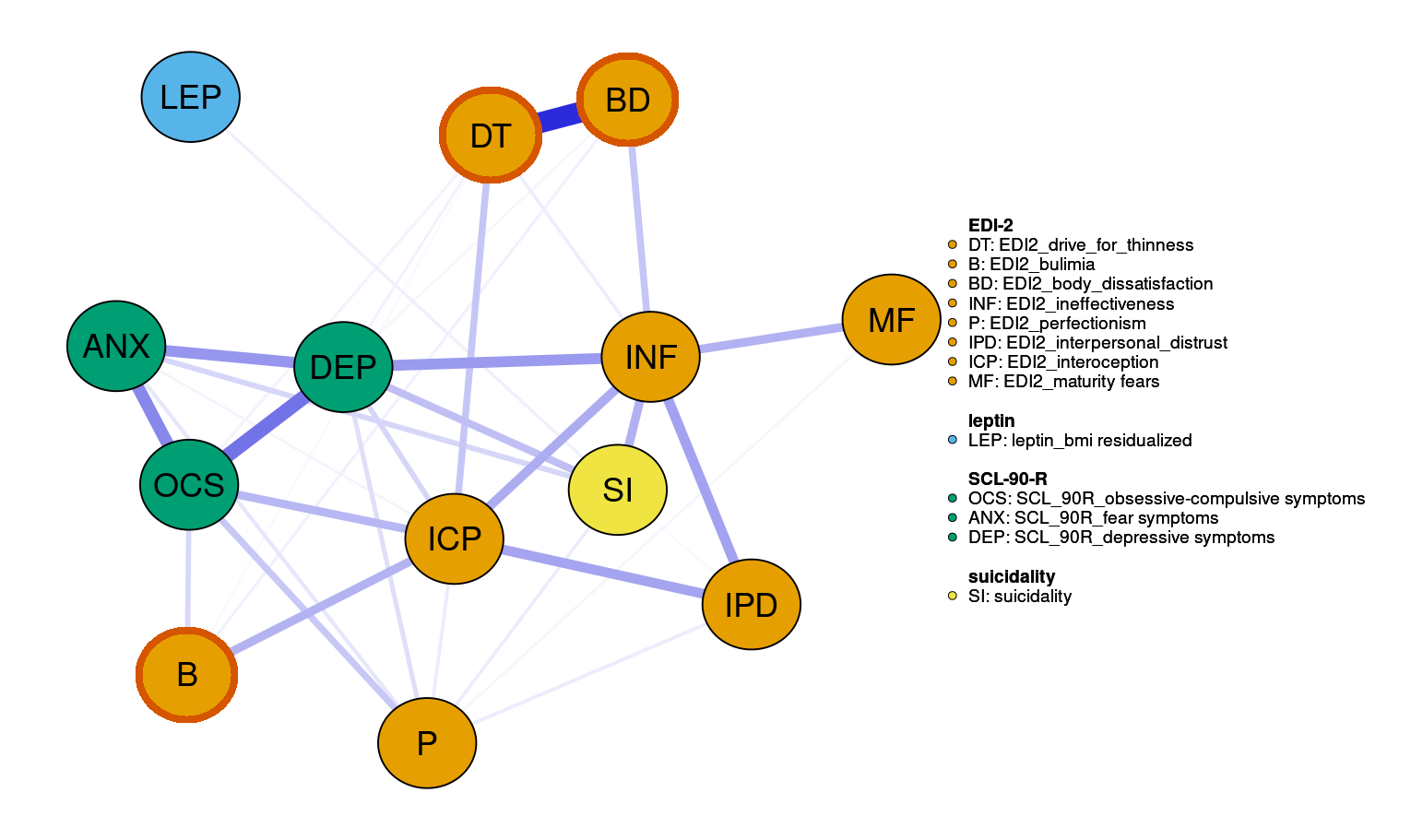
*

Figure S8

Estimated network of total patient population (n=229) at timepoint 1 including leptin adjusted for BMI. Green nodes: Subscales of Symptom Checklist-90-Revised. Orange nodes: Subscales of Eating Disorder Inventory-2 (EDI-2). Darker frame around orange nodes: ED-specific symptoms (EDI-2). Blue lines: positive partial associations. Red lines: negative partial associations. Thickness and strength of lines: strength of the partial correlation. CS-coefficient strength: 0.75.

*Note.* SI, suicidal ideation; DEP, depressive symptom score of SCL-90-R (generated without item #15); ANX, anxiety symptom score of SCL-90-R; OCS, obsessive-compulsive symptom score of SCL-90-R; DT, EDI-2 scale drive for thinness; B, EDI-2 scale bulimia; BD, EDI-2 scale body dissatisfaction; INF, EDI-2 scale ineffectiveness; P, EDI-2 scale perfectionism; IPD, EDI-2 scale interpersonal distrust; ICP, EDI-2 scale interoception; MF, EDI-2 scale maturity fears; LEP, leptin.

*
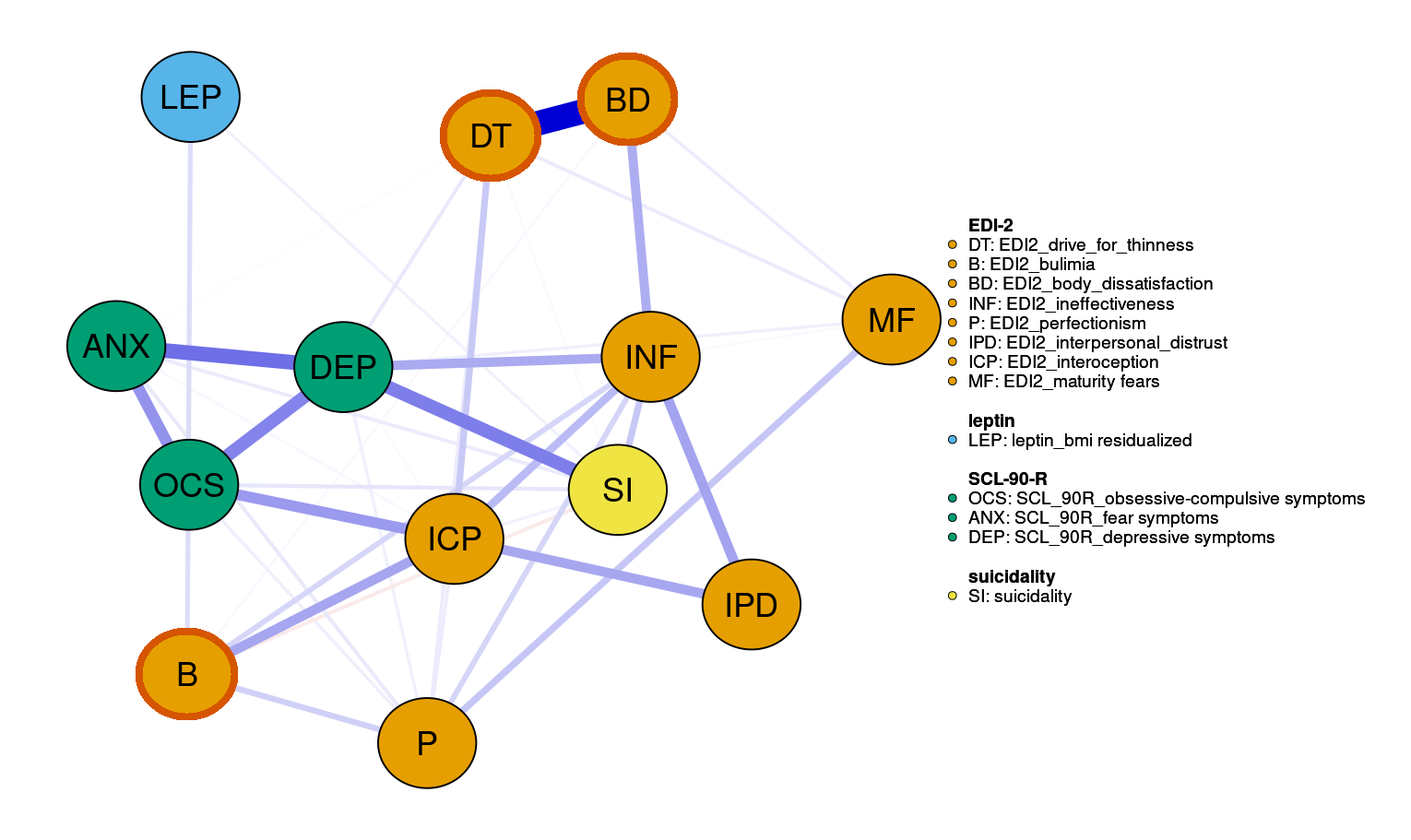
*

Figure S9

Estimated network of total patient population (n=150) at timepoint 2 including leptin adjusted for BMI. Green nodes: Subscales of Symptom Checklist-90-Revised. Orange nodes: Subscales of Eating Disorder Inventory-2 (EDI-2). Darker frame around orange nodes: ED-specific symptoms (EDI-2). Blue lines: positive partial associations. Red lines: negative partial associations. Thickness and strength of lines: strength of the partial correlation. CS-coefficient strength: 0.67.

*Note.* SI, suicidal ideation; DEP, depressive symptom score of SCL-90-R (generated without item #15); ANX, anxiety symptom score of SCL-90-R; OCS, obsessive-compulsive symptom score of SCL-90-R; DT, EDI-2 scale drive for thinness; B, EDI-2 scale bulimia; BD, EDI-2 scale body dissatisfaction; INF, EDI-2 scale ineffectiveness; P, EDI-2 scale perfectionism; IPD, EDI-2 scale interpersonal distrust; ICP, EDI-2 scale interoception; MF, EDI-2 scale maturity fears; LEP, leptin.

*
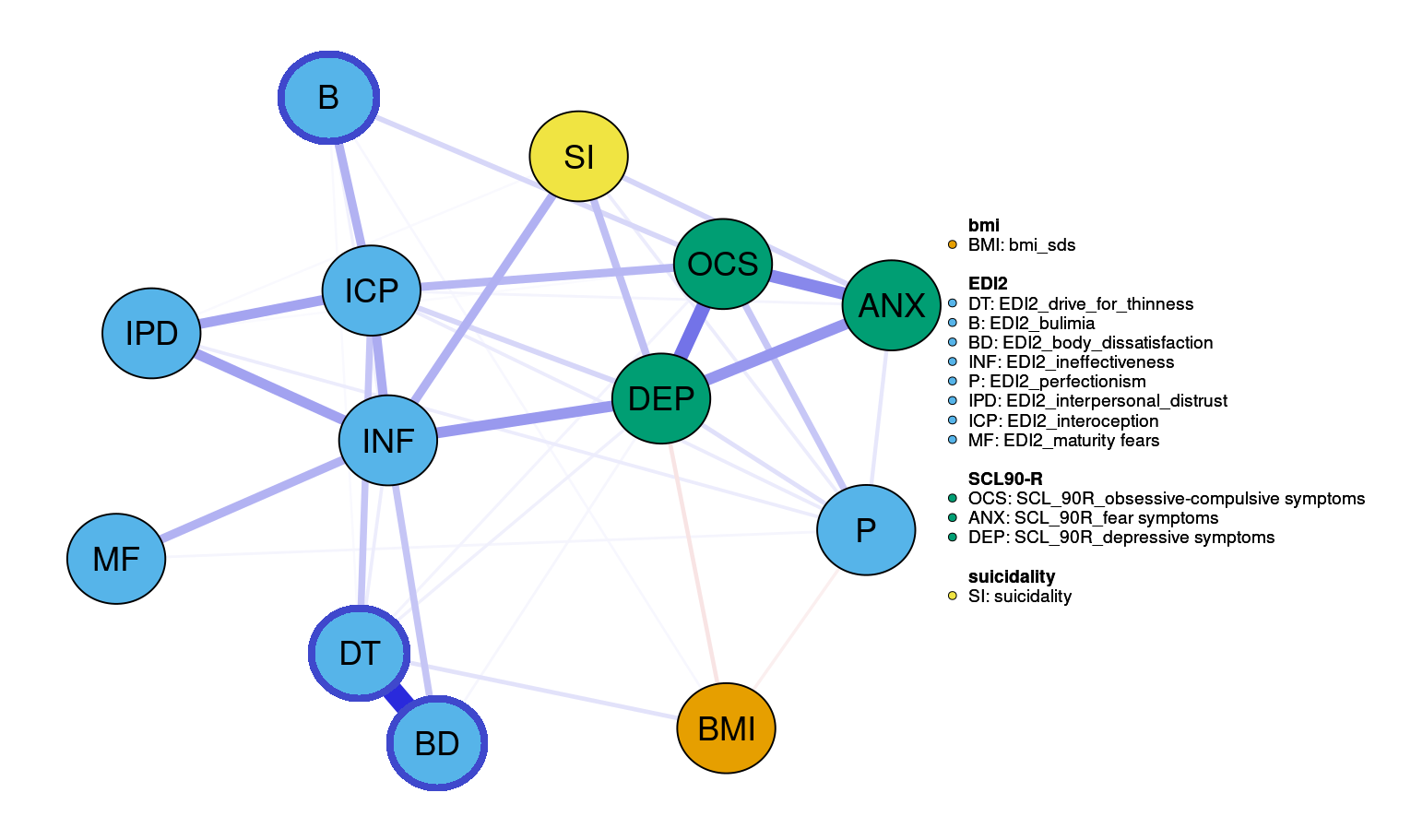
*

Figure S10

Estimated network of total patient population (n=229) at timepoint 1 including BMI (without leptin). Green nodes: Subscales of Symptom Checklist-90-Revised. Blue nodes: Subscales of Eating Disorder Inventory-2 (EDI-2). Darker frame around blue nodes: ED-specific symptoms (EDI-2). Blue lines: positive partial associations. Red lines: negative partial associations. Thickness and strength of lines: strength of the partial correlation. CS-coefficient strength: 0.75.

*Note.* SI, suicidal ideation; DEP, depressive symptom score of SCL-90-R (generated without item #15); ANX, anxiety symptom score of SCL-90-R; OCS, obsessive-compulsive symptom score of SCL-90-R; DT, EDI-2 scale drive for thinness; B, EDI-2 scale bulimia; BD, EDI-2 scale body dissatisfaction; INF, EDI-2 scale ineffectiveness; P, EDI-2 scale perfectionism; IPD, EDI-2 scale interpersonal distrust; ICP, EDI-2 scale interoception; MF, EDI-2 scale maturity fears; BMI, body mass index.


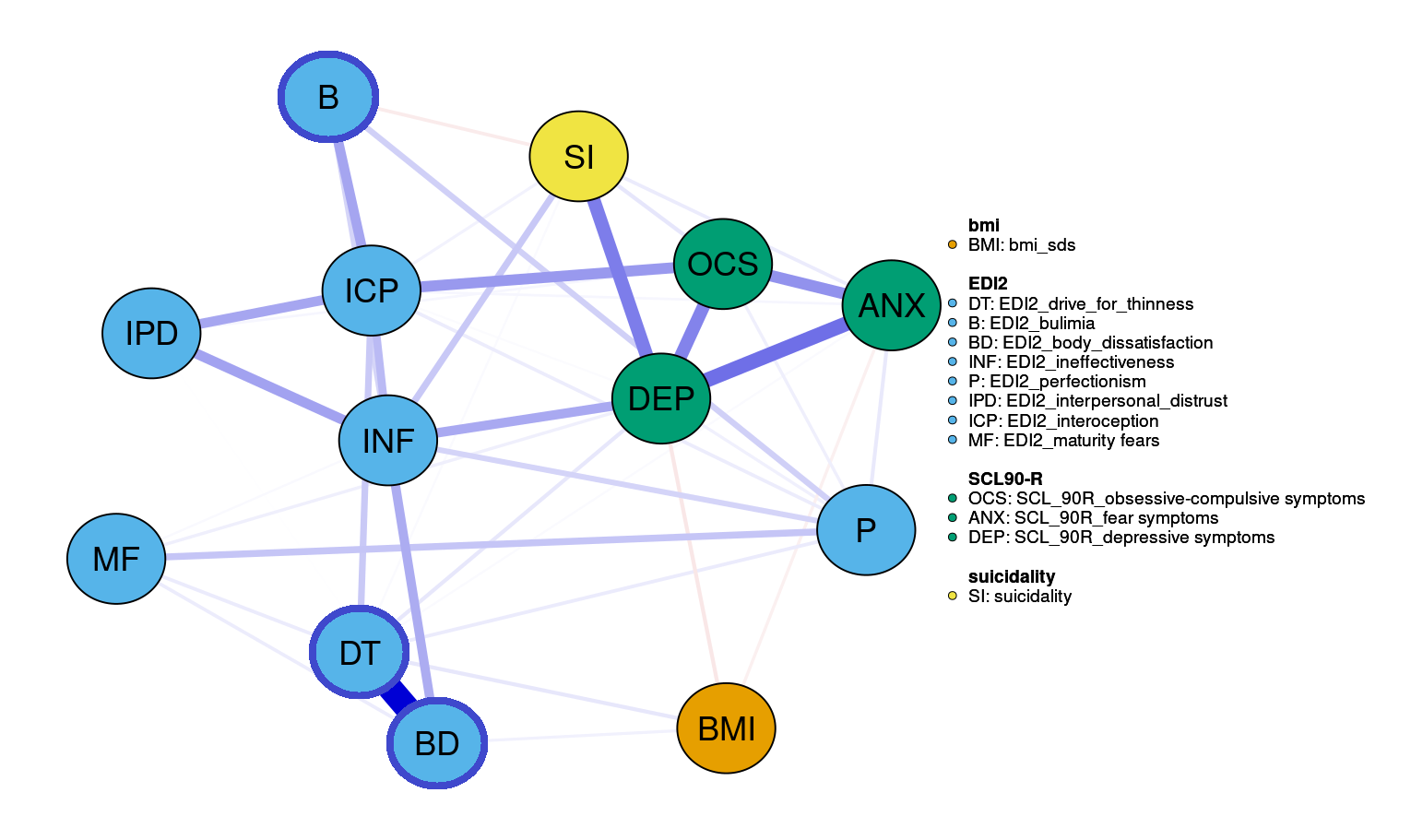


Figure S11

Estimated network of total patient population (n=150) at timepoint 2 including BMI (without leptin). Green nodes: Subscales of Symptom Checklist-90-Revised. Blue nodes: Subscales of Eating Disorder Inventory-2 (EDI-2). Darker frame around blue nodes: ED-specific symptoms (EDI-2). Blue lines: positive partial associations. Red lines: negative partial associations. Thickness and strength of lines: strength of the partial correlation. CS-coefficient strength: 0.67.

*Note.* SI, suicidal ideation; DEP, depressive symptom score of SCL-90-R (generated without item #15); ANX, anxiety symptom score of SCL-90-R; OCS, obsessive-compulsive symptom score of SCL-90-R; DT, EDI-2 scale drive for thinness; B, EDI-2 scale bulimia; BD, EDI-2 scale body dissatisfaction; INF, EDI-2 scale ineffectiveness; P, EDI-2 scale perfectionism; IPD, EDI-2 scale interpersonal distrust; ICP, EDI-2 scale interoception; MF, EDI-2 scale maturity fears; BMI, body mass index.


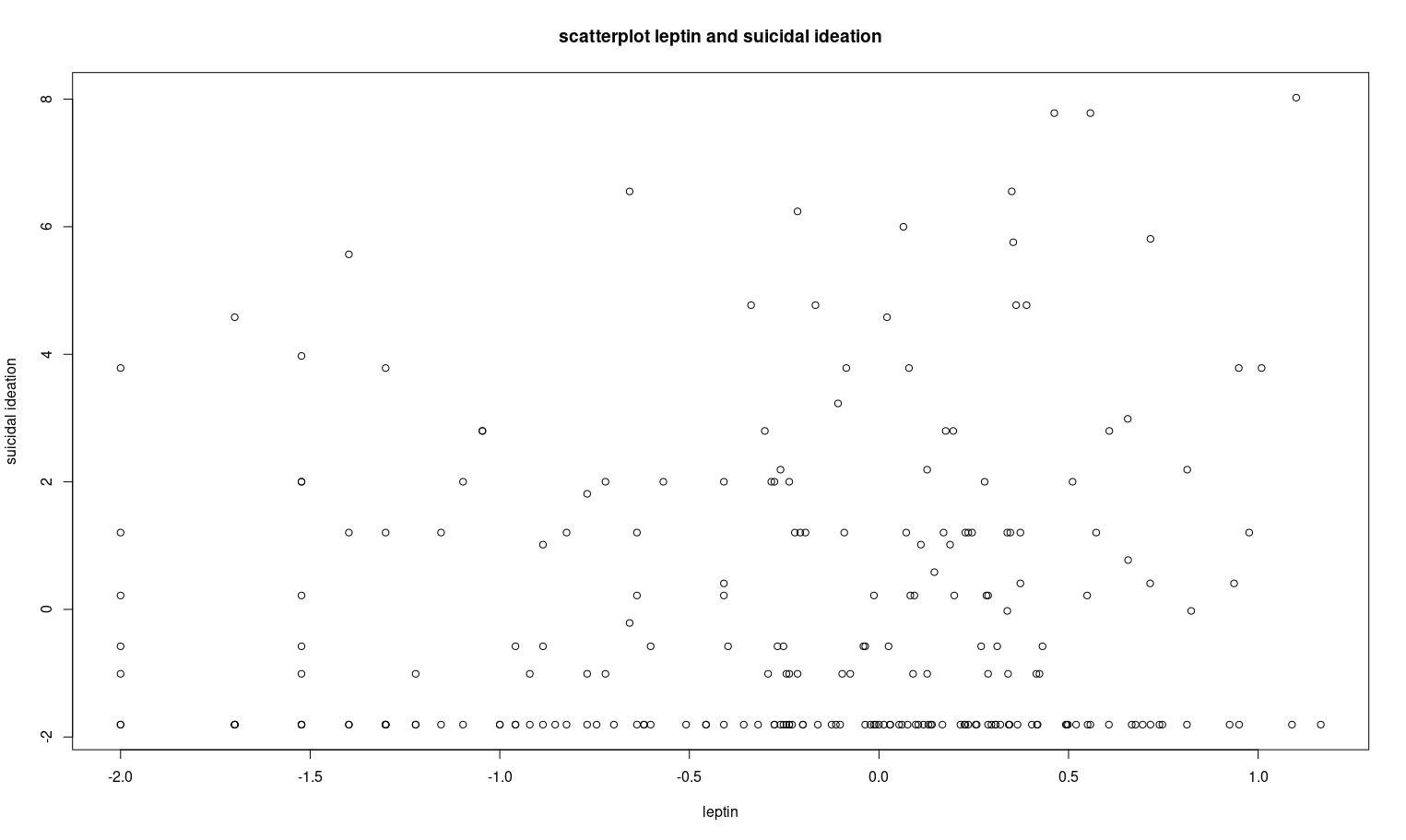


Figure S12

Scatterplot depicting the relationship between suicidal ideation (SI) and leptin levels.

*Note.* Suicidal ideation scores are z-scaled, and leptin values are log10-transformed.

*
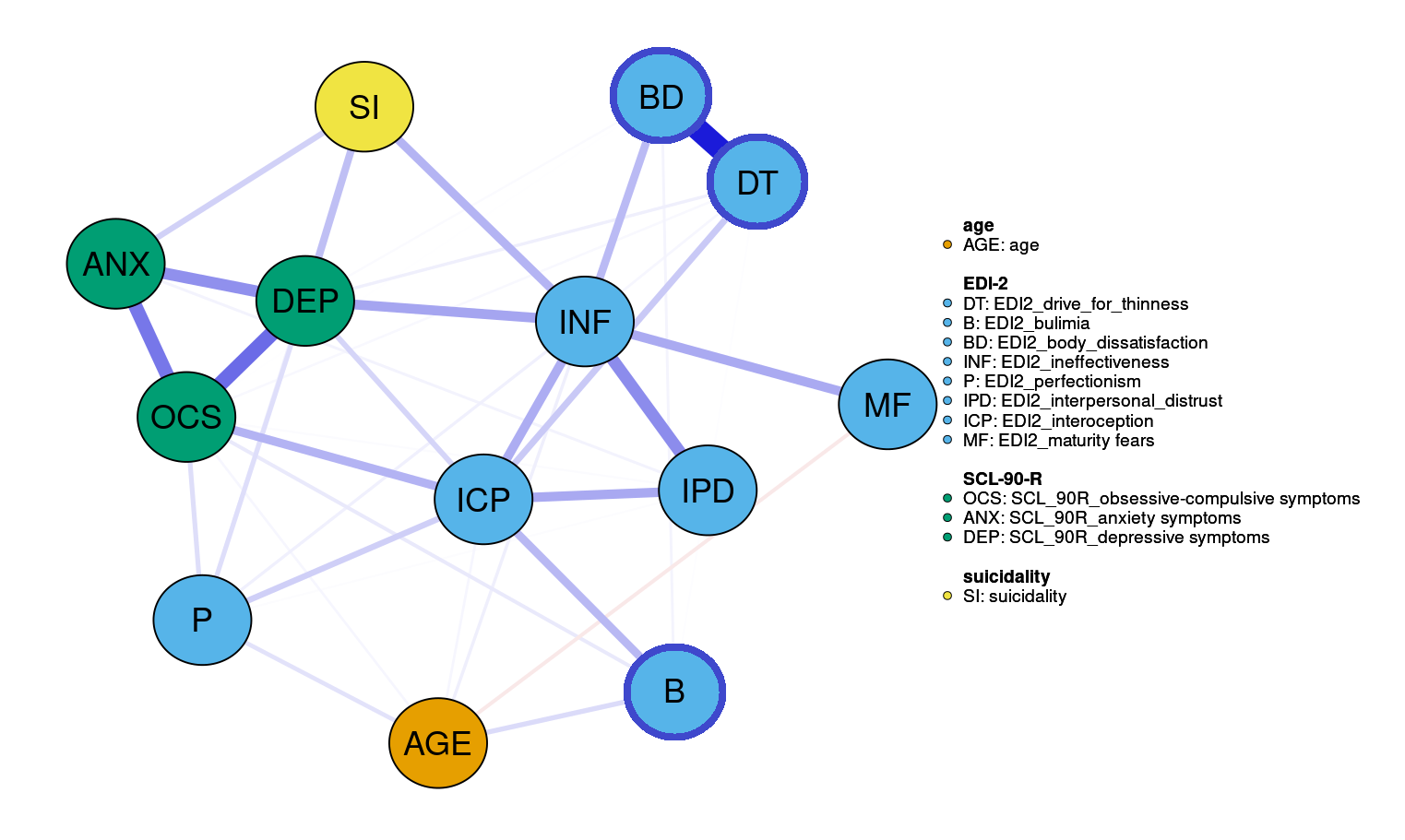
*

Figure S13

Estimated network of total patient population (n=313) at timepoint 1 including age in the original network. Green nodes: Subscales of Symptom Checklist-90-Revised. Blue nodes: Subscales of Eating Disorder Inventory-2 (EDI-2). Darker frame around blue nodes: ED-specific symptoms (EDI-2). Blue lines: positive partial associations. Red lines: negative partial associations. Thickness and strength of lines: strength of the partial correlation. CS-coefficient strength: 0.75.

*Note.* SI, suicidal ideation; DEP, depressive symptom score of SCL-90-R (generated without item #15); ANX, anxiety symptom score of SCL-90-R; OCS, obsessive-compulsive symptom score of SCL-90-R; DT, EDI-2 scale drive for thinness; B, EDI-2 scale bulimia; BD, EDI-2 scale body dissatisfaction; INF, EDI-2 scale ineffectiveness; P, EDI-2 scale perfectionism; IPD, EDI-2 scale interpersonal distrust; ICP, EDI-2 scale interoception; MF, EDI-2 scale maturity fears.

*
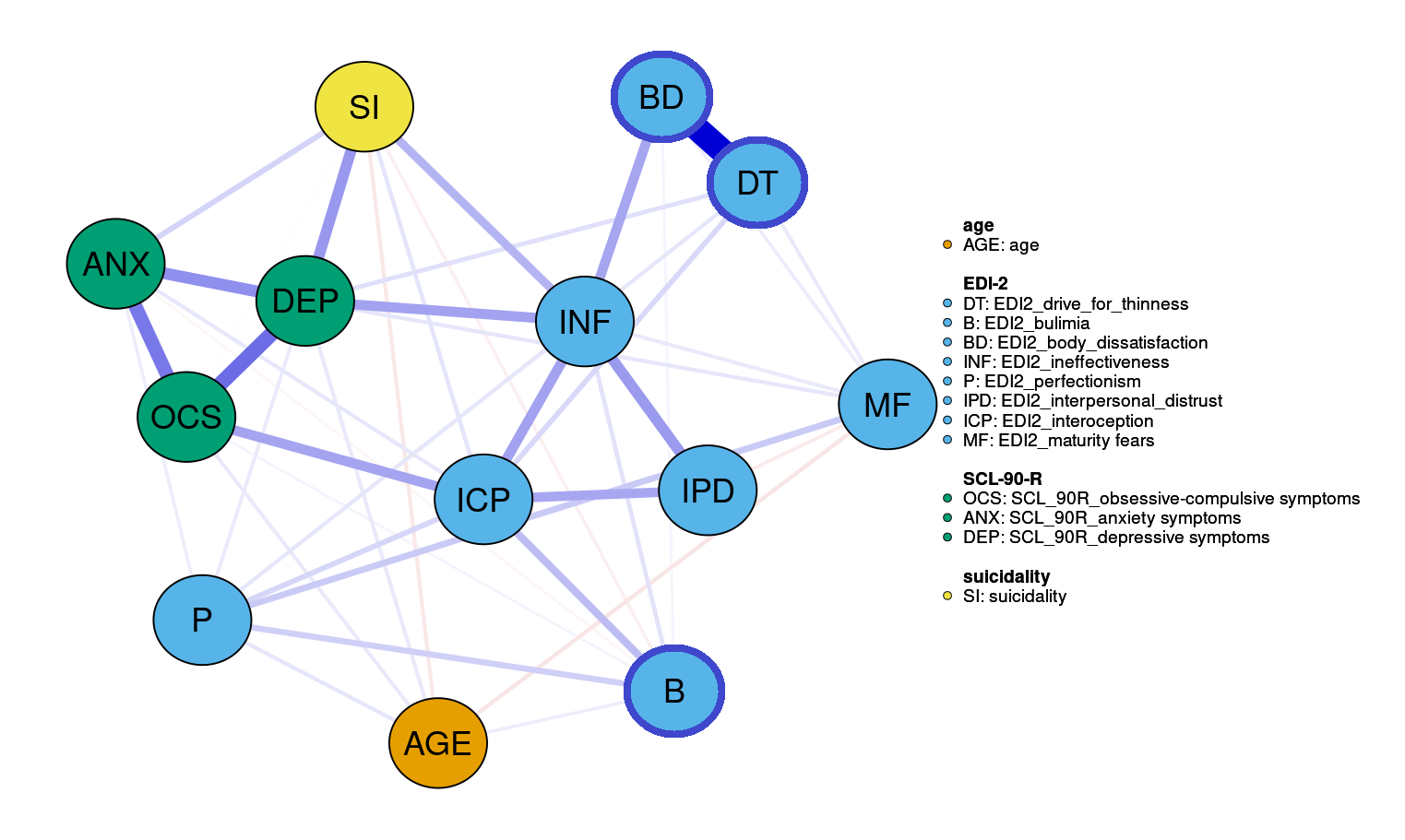
*

Figure S14

Estimated network of total patient population (n=217) at timepoint 2 including age in the original network. Green nodes: Subscales of Symptom Checklist-90-Revised. Blue nodes: Subscales of Eating Disorder Inventory-2 (EDI-2). Darker frame around blue nodes: ED-specific symptoms (EDI-2). Blue lines: positive partial associations. Red lines: negative partial associations. Thickness and strength of lines: strength of the partial correlation. CS-coefficient strength: 0.75.

*Note.* SI, suicidal ideation; DEP, depressive symptom score of SCL-90-R (generated without item #15); ANX, anxiety symptom score of SCL-90-R; OCS, obsessive-compulsive symptom score of SCL-90-R; DT, EDI-2 scale drive for thinness; B, EDI-2 scale bulimia; BD, EDI-2 scale body dissatisfaction; INF, EDI-2 scale ineffectiveness; P, EDI-2 scale perfectionism; IPD, EDI-2 scale interpersonal distrust; ICP, EDI-2 scale interoception; MF, EDI-2 scale maturity fears.

*
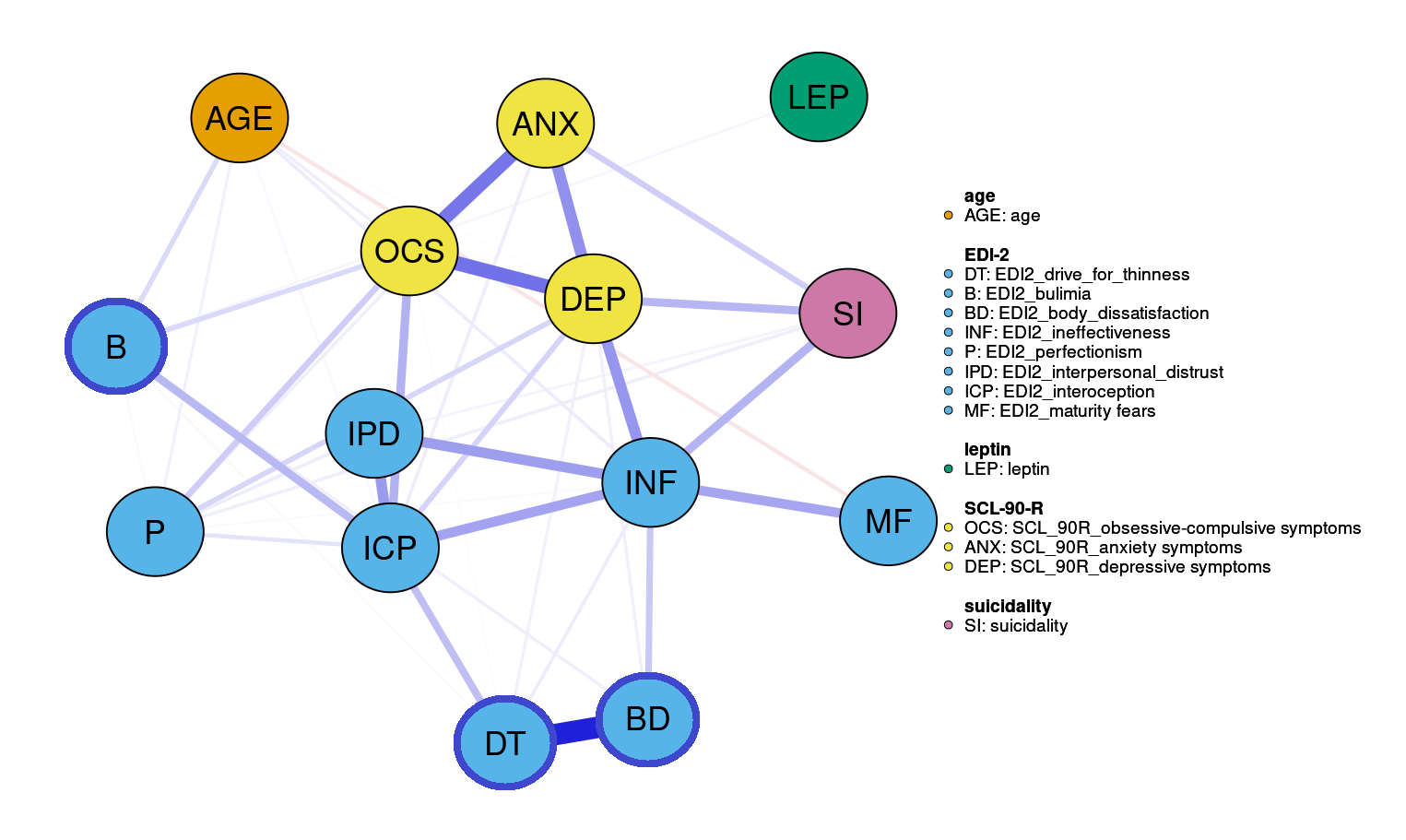
*

Figure S15

Estimated network of total patient population (n=229) at timepoint 1 including age and leptin in the original network. Yellow nodes: Subscales of Symptom Checklist-90-Revised. Blue nodes: Subscales of Eating Disorder Inventory-2 (EDI-2). Darker frame around blue nodes: ED-specific symptoms (EDI-2). Blue lines: positive partial associations. Red lines: negative partial associations. Thickness and strength of lines: strength of the partial correlation. CS-coefficient strength: 0.75.

*Note.* SI, suicidal ideation; DEP, depressive symptom score of SCL-90-R (generated without item #15); ANX, anxiety symptom score of SCL-90-R; OCS, obsessive-compulsive symptom score of SCL-90-R; DT, EDI-2 scale drive for thinness; B, EDI-2 scale bulimia; BD, EDI-2 scale body dissatisfaction; INF, EDI-2 scale ineffectiveness; P, EDI-2 scale perfectionism; IPD, EDI-2 scale interpersonal distrust; ICP, EDI-2 scale interoception; MF, EDI-2 scale maturity fears; LEP, leptin.

*
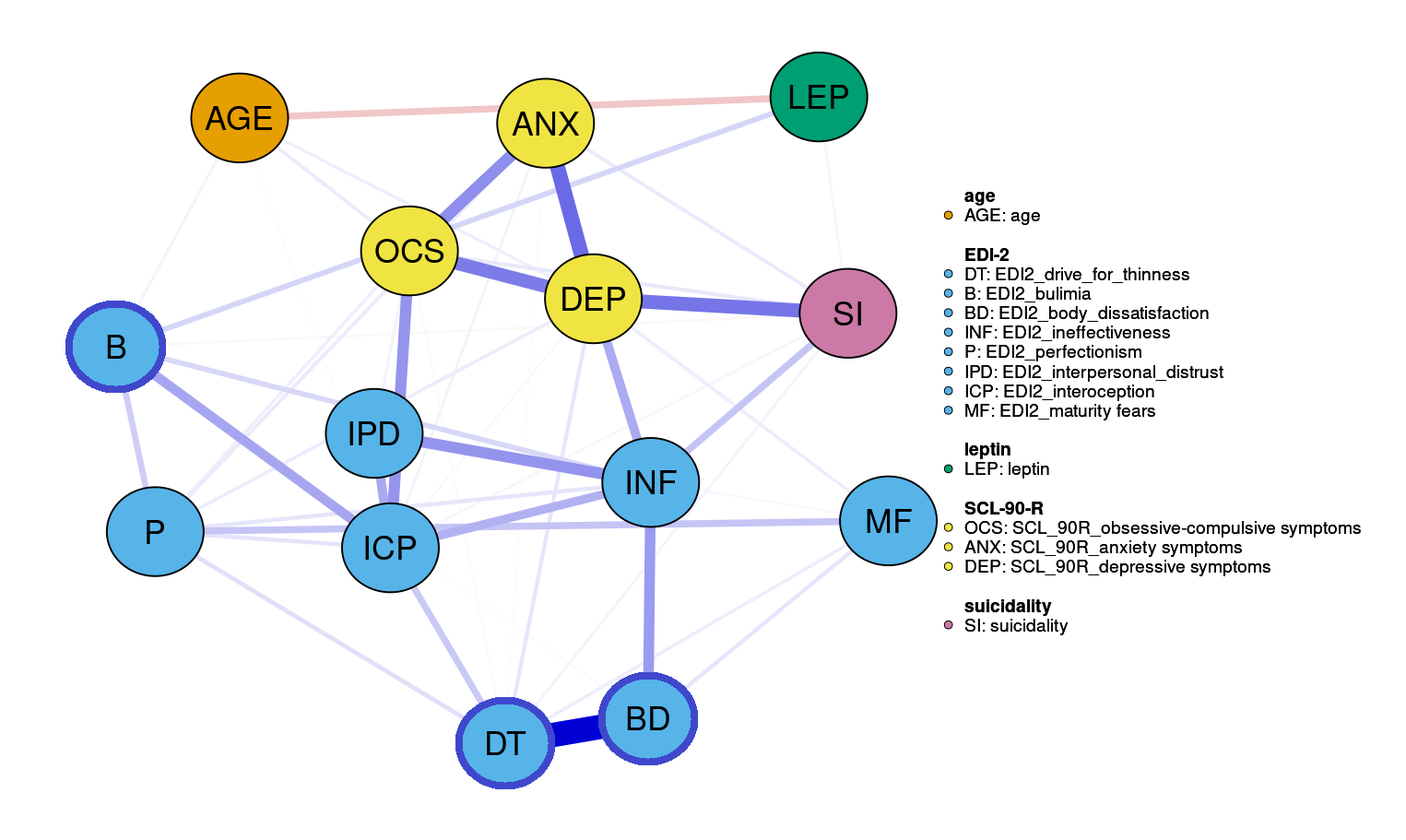
*

Figure S16

Estimated network of total patient population (n=150) at timepoint 2 including age and leptin in the original network. Yellow nodes: Subscales of Symptom Checklist-90-Revised. Blue nodes: Subscales of Eating Disorder Inventory-2 (EDI-2). Darker frame around blue nodes: ED-specific symptoms (EDI-2). Blue lines: positive partial associations. Red lines: negative partial associations. Thickness and strength of lines: strength of the partial correlation. CS-coefficient strength: 0.59.

*Note.* SI, suicidal ideation; DEP, depressive symptom score of SCL-90-R (generated without item #15); ANX, anxiety symptom score of SCL-90-R; OCS, obsessive-compulsive symptom score of SCL-90-R; DT, EDI-2 scale drive for thinness; B, EDI-2 scale bulimia; BD, EDI-2 scale body dissatisfaction; INF, EDI-2 scale ineffectiveness; P, EDI-2 scale perfectionism; IPD, EDI-2 scale interpersonal distrust; ICP, EDI-2 scale interoception; MF, EDI-2 scale maturity fears; LEP, leptin.

*
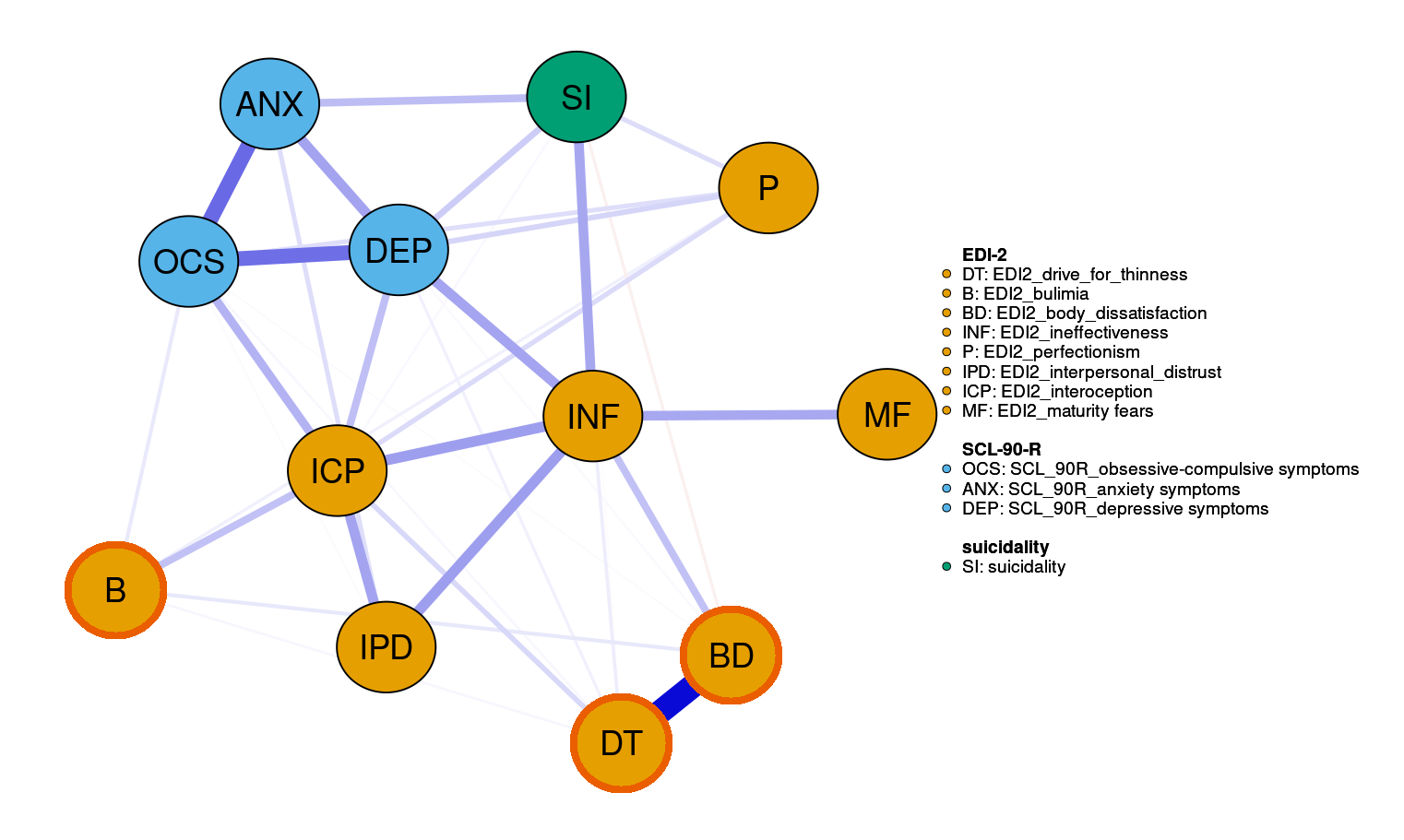
*

Figure S17

Estimated network of patient population below the age of 18 years (n=253) at timepoint 1. Blue nodes: Subscales of Symptom Checklist-90-Revised. Orange nodes: Subscales of Eating Disorder Inventory-2 (EDI-2). Darker frame around orange nodes: ED-specific symptoms (EDI-2). Blue lines: positive partial associations. Red lines: negative partial associations. Thickness and strength of lines: strength of the partial correlation. CS-coefficient strength: 0.75.

*Note.* SI, suicidal ideation; DEP, depressive symptom score of SCL-90-R (generated without item #15); ANX, anxiety symptom score of SCL-90-R; OCS, obsessive-compulsive symptom score of SCL-90-R; DT, EDI-2 scale drive for thinness; B, EDI-2 scale bulimia; BD, EDI-2 scale body dissatisfaction; INF, EDI-2 scale ineffectiveness; P, EDI-2 scale perfectionism; IPD, EDI-2 scale interpersonal distrust; ICP, EDI-2 scale interoception; MF, EDI-2 scale maturity fears.

*
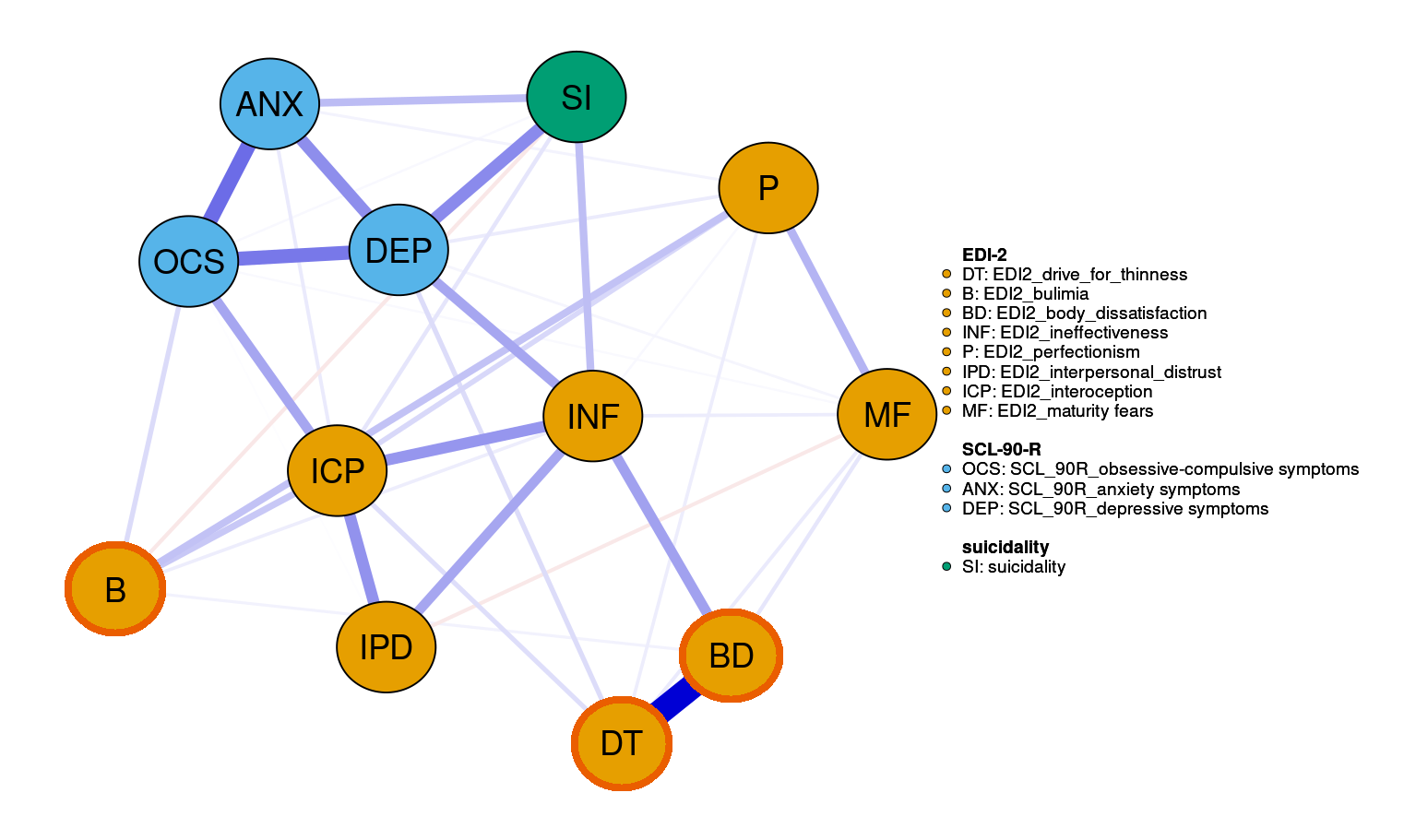
*

Figure S18

Estimated network of patient population below the age of 18 years (n=183) at timepoint 2. Blue nodes: Subscales of Symptom Checklist-90-Revised. Orange nodes: Subscales of Eating Disorder Inventory-2 (EDI-2). Darker frame around orange nodes: ED-specific symptoms (EDI-2). Blue lines: positive partial associations. Red lines: negative partial associations. Thickness and strength of lines: strength of the partial correlation. CS-coefficient strength: 0.75.

*Note.* SI, suicidal ideation; DEP, depressive symptom score of SCL-90-R (generated without item #15); ANX, anxiety symptom score of SCL-90-R; OCS, obsessive-compulsive symptom score of SCL-90-R; DT, EDI-2 scale drive for thinness; B, EDI-2 scale bulimia; BD, EDI-2 scale body dissatisfaction; INF, EDI-2 scale ineffectiveness; P, EDI-2 scale perfectionism; IPD, EDI-2 scale interpersonal distrust; ICP, EDI-2 scale interoception; MF, EDI-2 scale maturity fears.

*
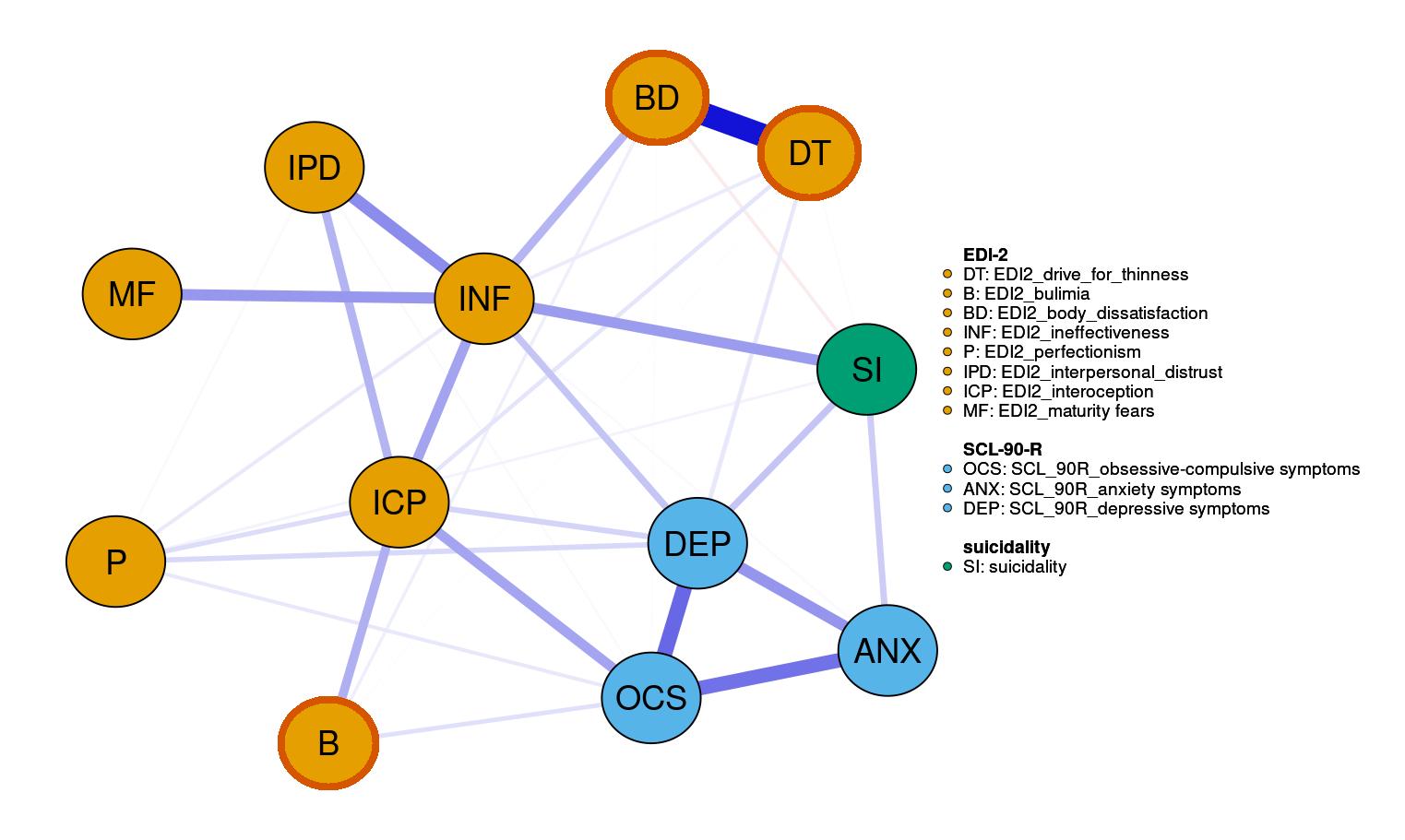
*

Figure S19

Estimated network of patient population without psychoactive medication (n=298) at timepoint 1. Blue nodes: Subscales of Symptom Checklist-90-Revised. Orange nodes: Subscales of Eating Disorder Inventory-2 (EDI-2). Darker frame around orange nodes: ED-specific symptoms (EDI-2). Blue lines: positive partial associations. Red lines: negative partial associations. Thickness and strength of lines: strength of the partial correlation. CS-coefficient strength: 0.75.

*Note.* SI, suicidal ideation; DEP, depressive symptom score of SCL-90-R (generated without item #15); ANX, anxiety symptom score of SCL-90-R; OCS, obsessive-compulsive symptom score of SCL-90-R; DT, EDI-2 scale drive for thinness; B, EDI-2 scale bulimia; BD, EDI-2 scale body dissatisfaction; INF, EDI-2 scale ineffectiveness; P, EDI-2 scale perfectionism; IPD, EDI-2 scale interpersonal distrust; ICP, EDI-2 scale interoception; MF, EDI-2 scale maturity fears.

*
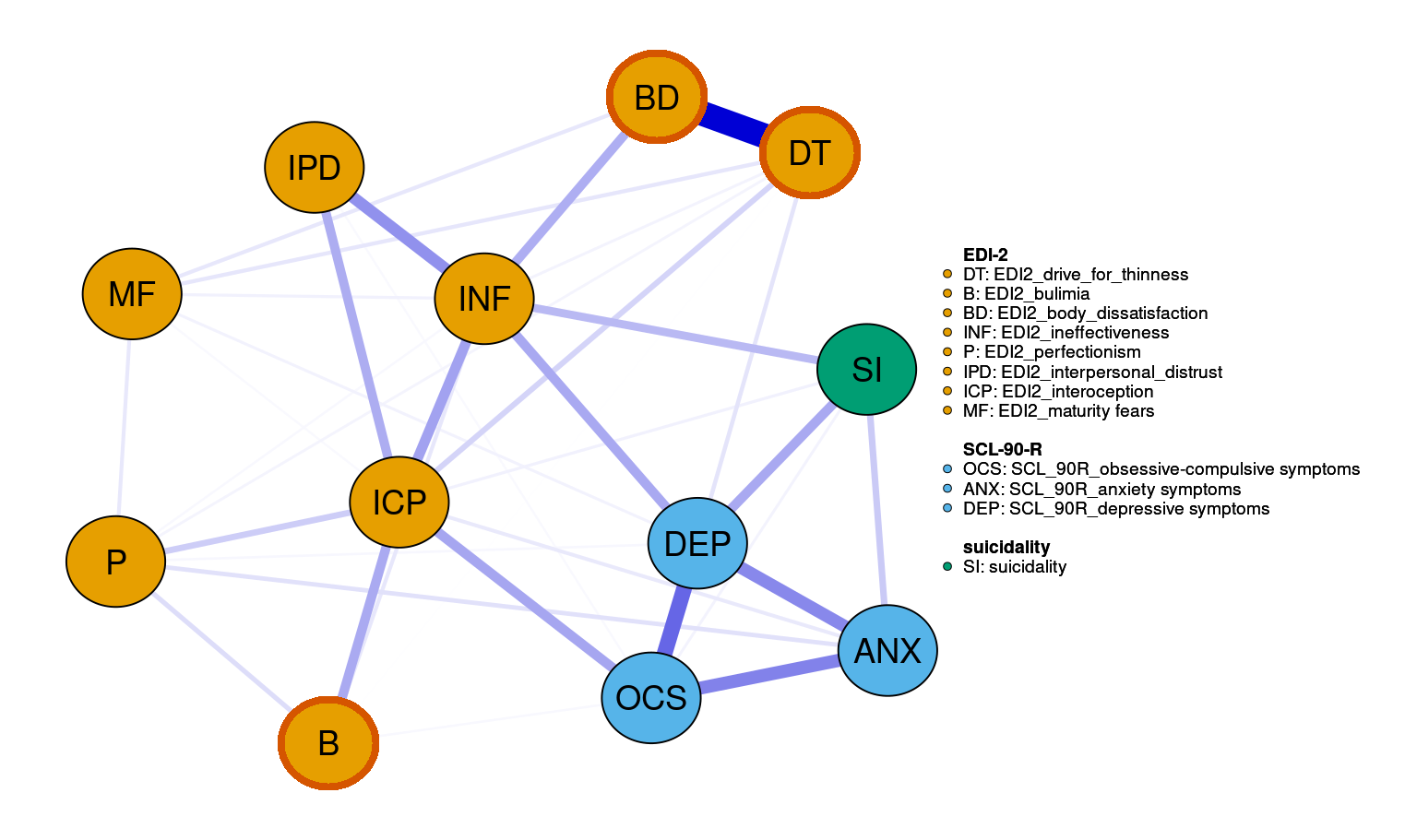
*

Figure S20

Estimated network of patient population without psychoactive medication (n=208) at timepoint 2. Blue nodes: Subscales of Symptom Checklist-90-Revised. Orange nodes: Subscales of Eating Disorder Inventory-2 (EDI-2). Darker frame around orange nodes: ED-specific symptoms (EDI-2). Blue lines: positive partial associations. Red lines: negative partial associations. Thickness and strength of lines: strength of the partial correlation. CS-coefficient strength: 0.75.

*Note.* SI, suicidal ideation; DEP, depressive symptom score of SCL-90-R (generated without item #15); ANX, anxiety symptom score of SCL-90-R; OCS, obsessive-compulsive symptom score of SCL-90-R; DT, EDI-2 scale drive for thinness; B, EDI-2 scale bulimia; BD, EDI-2 scale body dissatisfaction; INF, EDI-2 scale ineffectiveness; P, EDI-2 scale perfectionism; IPD, EDI-2 scale interpersonal distrust; ICP, EDI-2 scale interoception; MF, EDI-2 scale maturity fears.

*
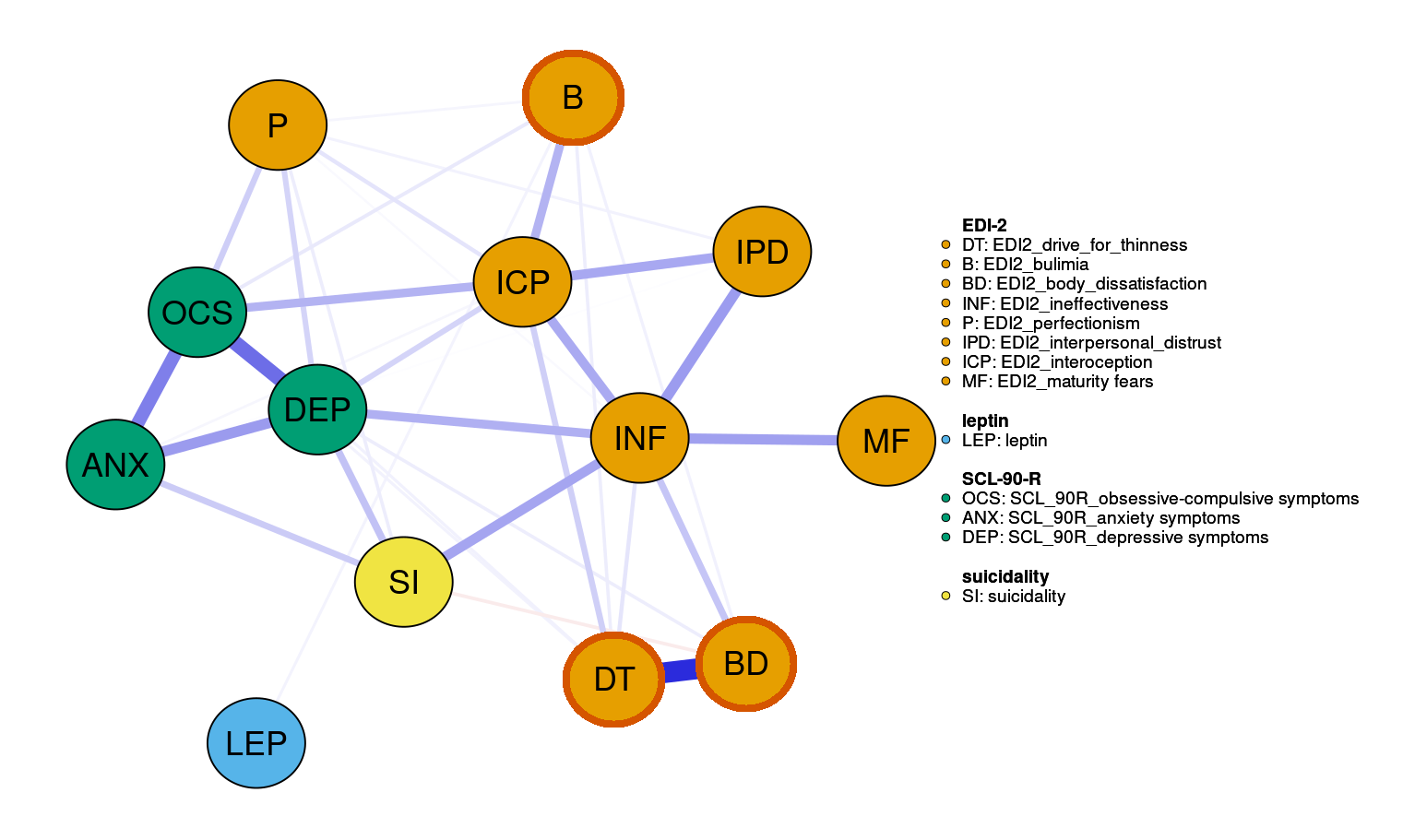
*

Figure S21

Estimated network of patient population without psychoactive medication (n=221) at timepoint 1 including leptin. Green nodes: Subscales of Symptom Checklist-90-Revised. Orange nodes: Subscales of Eating Disorder Inventory-2 (EDI-2). Darker frame around orange nodes: ED-specific symptoms (EDI-2). Blue lines: positive partial associations. Red lines: negative partial associations. Thickness and strength of lines: strength of the partial correlation. CS-coefficient strength: 0.75.

*Note.* SI, suicidal ideation; DEP, depressive symptom score of SCL-90-R (generated without item #15); ANX, anxiety symptom score of SCL-90-R; OCS, obsessive-compulsive symptom score of SCL-90-R; DT, EDI-2 scale drive for thinness; B, EDI-2 scale bulimia; BD, EDI-2 scale body dissatisfaction; INF, EDI-2 scale ineffectiveness; P, EDI-2 scale perfectionism; IPD, EDI-2 scale interpersonal distrust; ICP, EDI-2 scale interoception; MF, EDI-2 scale maturity fears; LEP, leptin.

*
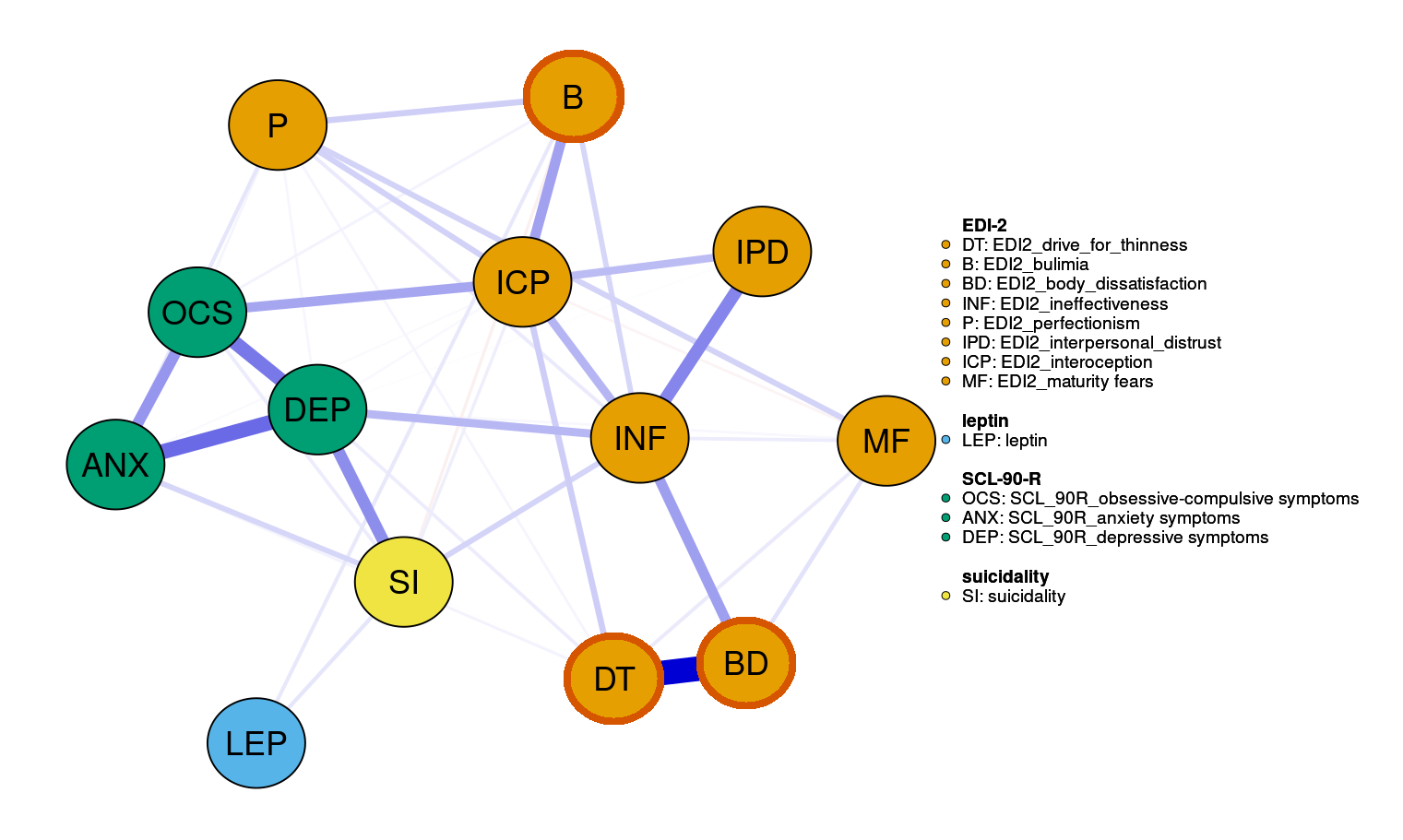
*

Figure S22

Estimated network of patient population without psychoactive medication (n=142) at timepoint 2 including leptin. Green nodes: Subscales of Symptom Checklist-90-Revised. Orange nodes: Subscales of Eating Disorder Inventory-2 (EDI-2). Darker frame around orange nodes: ED-specific symptoms (EDI-2). Blue lines: positive partial associations. Red lines: negative partial associations. Thickness and strength of lines: strength of the partial correlation. CS-coefficient strength: 0.59.

*Note.* SI, suicidal ideation; DEP, depressive symptom score of SCL-90-R (generated without item #15); ANX, anxiety symptom score of SCL-90-R; OCS, obsessive-compulsive symptom score of SCL-90-R; DT, EDI-2 scale drive for thinness; B, EDI-2 scale bulimia; BD, EDI-2 scale body dissatisfaction; INF, EDI-2 scale ineffectiveness; P, EDI-2 scale perfectionism; IPD, EDI-2 scale interpersonal distrust; ICP, EDI-2 scale interoception; MF, EDI-2 scale maturity fears; LEP, leptin.

*
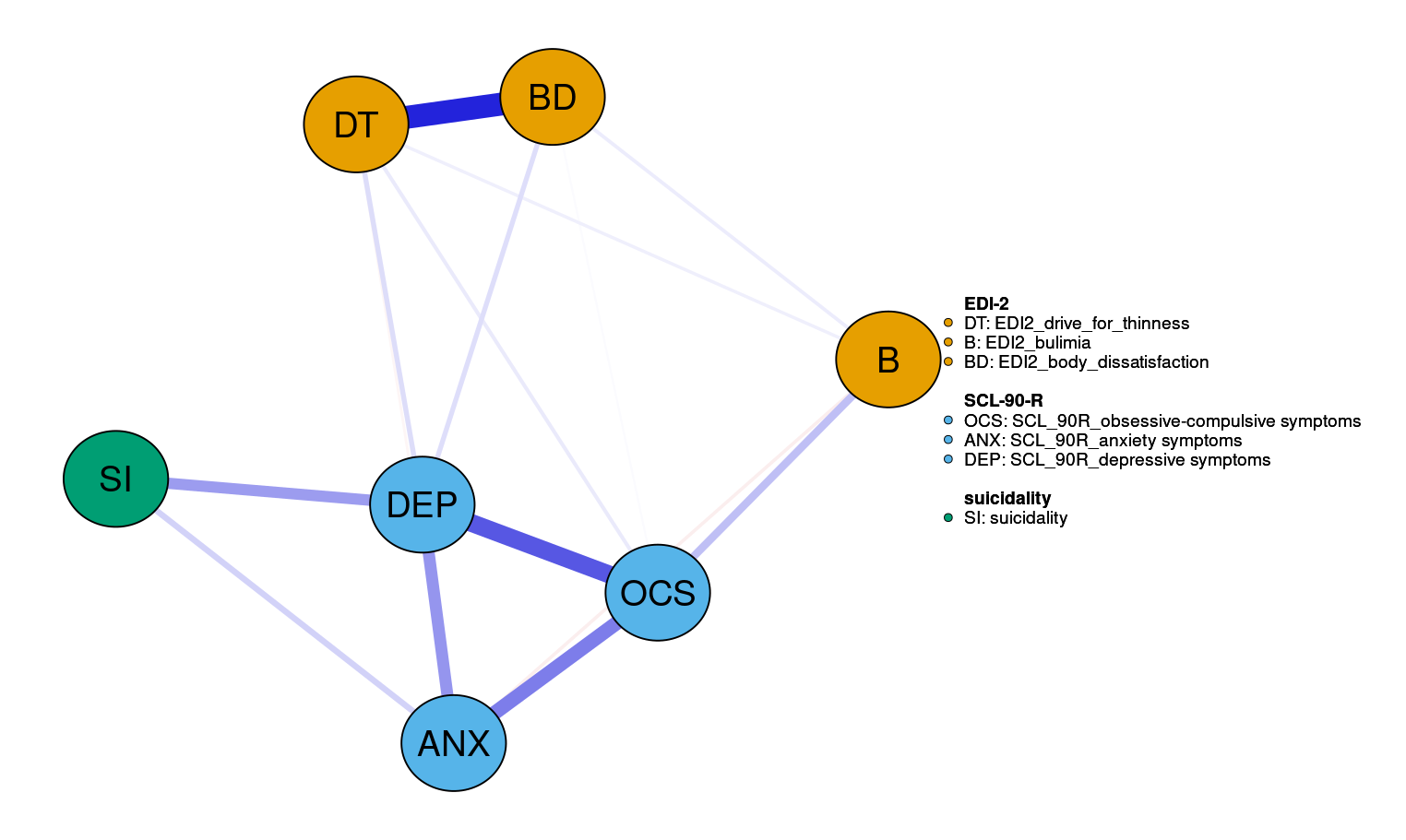
*

Figure S23

Estimated network of total patient population (n=313) at timepoint 1, including only ED-specific symptom nodes. Blue nodes: Subscales of Symptom Checklist-90-Revised. Orange nodes: Subscales of Eating Disorder Inventory-2 (EDI-2). Blue lines: positive partial associations. Red lines: negative partial associations. Thickness and strength of lines: strength of the partial correlation. CS-coefficient strength: 0.75.

*Note.* SI, suicidal ideation; DEP, depressive symptom score of SCL-90-R (generated without item #15); ANX, anxiety symptom score of SCL-90-R; OCS, obsessive-compulsive symptom score of SCL-90-R; DT, EDI-2 scale drive for thinness; B, EDI-2 scale bulimia; BD, EDI-2 scale body dissatisfaction.

*
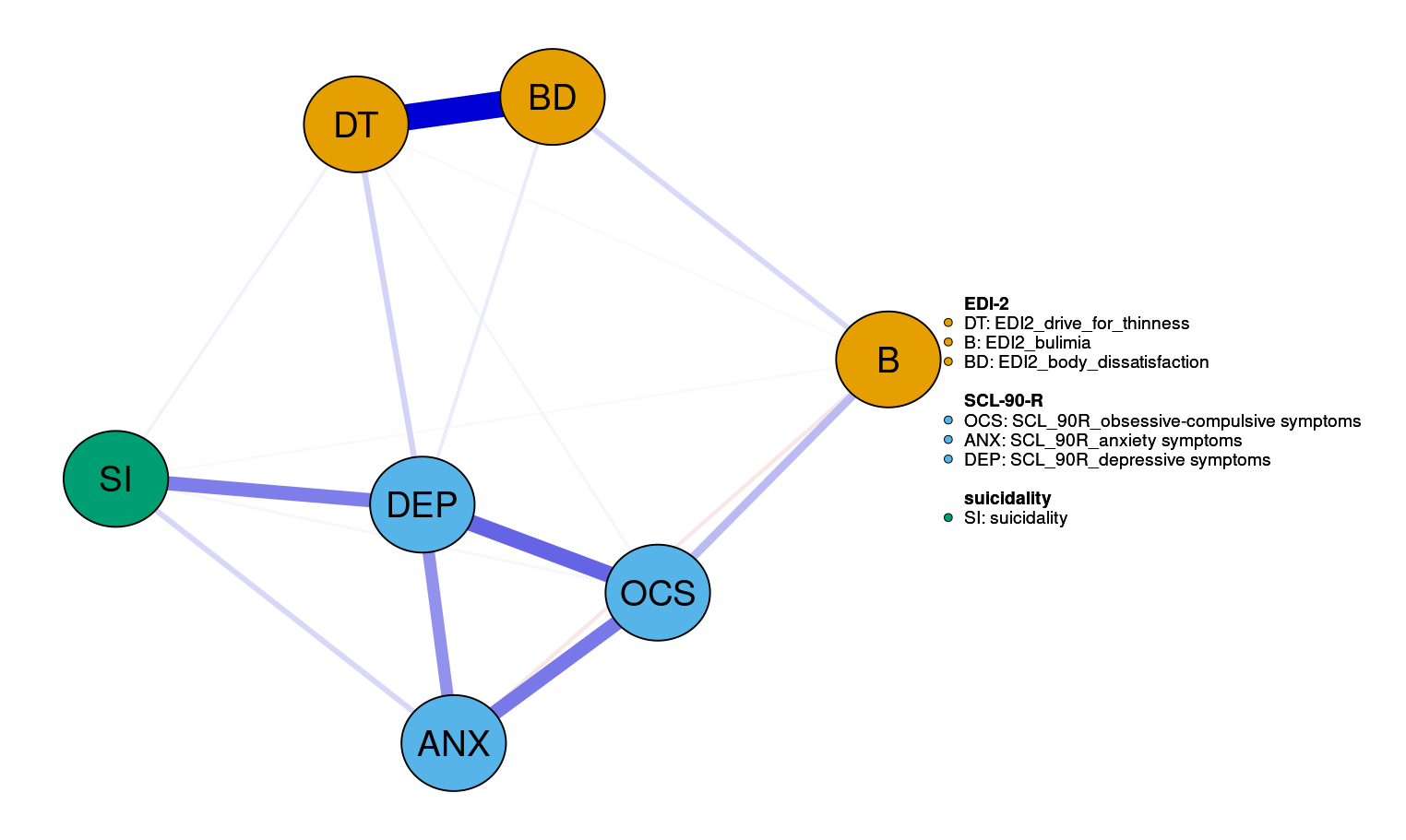
*

Figure S24

Estimated network of total patient population (n=217) at timepoint 2, including only ED-specific symptom nodes. Blue nodes: Subscales of Symptom Checklist-90-Revised. Orange nodes: Subscales of Eating Disorder Inventory-2 (EDI-2). Blue lines: positive partial associations. Red lines: negative partial associations. Thickness and strength of lines: strength of the partial correlation. CS-coefficient strength: 0.75.

*Note.* SI, suicidal ideation; DEP, depressive symptom score of SCL-90-R (generated without item #15); ANX, anxiety symptom score of SCL-90-R; OCS, obsessive-compulsive symptom score of SCL-90-R; DT, EDI-2 scale drive for thinness; B, EDI-2 scale bulimia; BD, EDI-2 scale body dissatisfaction.

*
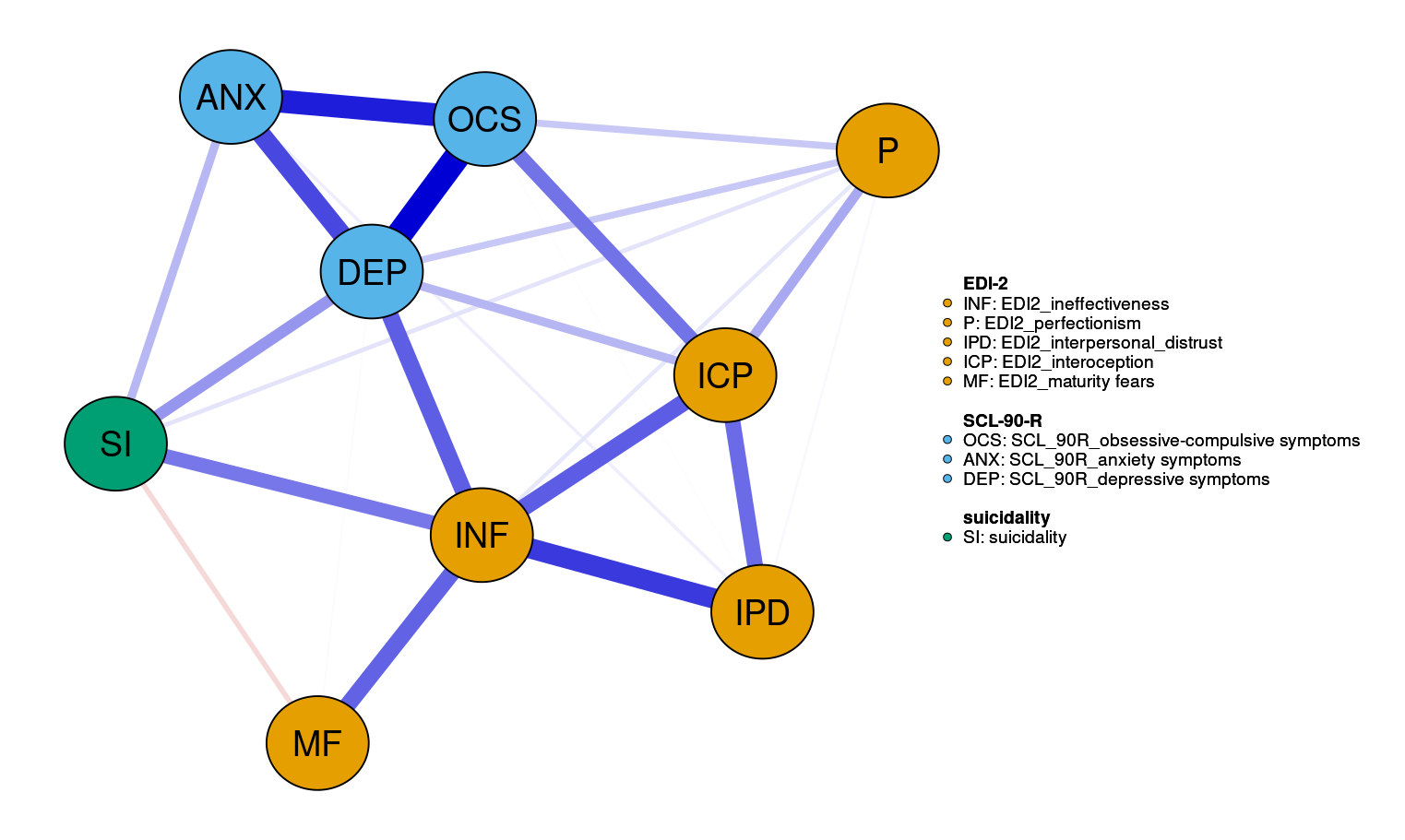
*

Figure S25

Estimated network of total patient population (n=313) at timepoint 1, including only ED-related symptom nodes. Blue nodes: Subscales of Symptom Checklist-90-Revised. Orange nodes: Subscales of Eating Disorder Inventory-2 (EDI-2). Blue lines: positive partial associations. Red lines: negative partial associations. Thickness and strength of lines: strength of the partial correlation. CS-coefficient strength: 0.75.

*Note.* SI, suicidal ideation; DEP, depressive symptom score of SCL-90-R (generated without item #15); ANX, anxiety symptom score of SCL-90-R; OCS, obsessive-compulsive symptom score of SCL-90-R; INF, EDI-2 scale ineffectiveness; P, EDI-2 scale perfectionism; IPD, EDI-2 scale interpersonal distrust; ICP, EDI-2 scale interoception; MF, EDI-2 scale maturity fears.

*
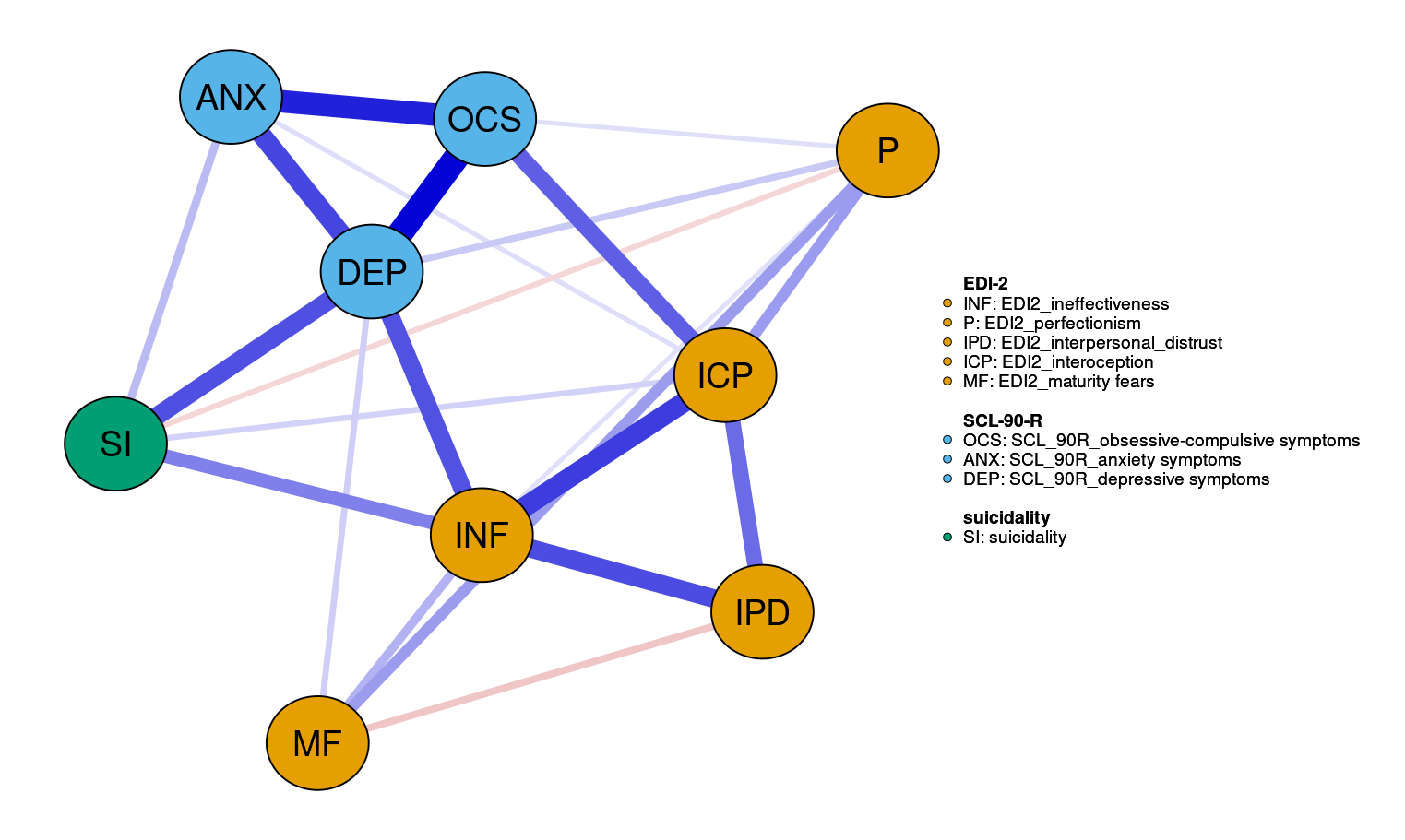
*

Figure S26

Estimated network of total patient population (n=217) at timepoint 2, including only ED-related symptom nodes. Blue nodes: Subscales of Symptom Checklist-90-Revised. Orange nodes: Subscales of Eating Disorder Inventory-2 (EDI-2). Darker frame around orange nodes: ED-specific symptoms (EDI-2). Blue lines: positive partial associations. Red lines: negative partial associations. Thickness and strength of lines: strength of the partial correlation. CS-coefficient strength: 0.75.

*Note.* SI, suicidal ideation; DEP, depressive symptom score of SCL-90-R (generated without item #15); ANX, anxiety symptom score of SCL-90-R; OCS, obsessive-compulsive symptom score of SCL-90-R; INF, EDI-2 scale ineffectiveness; P, EDI-2 scale perfectionism; IPD, EDI-2 scale interpersonal distrust; ICP, EDI-2 scale interoception; MF, EDI-2 scale maturity fears.


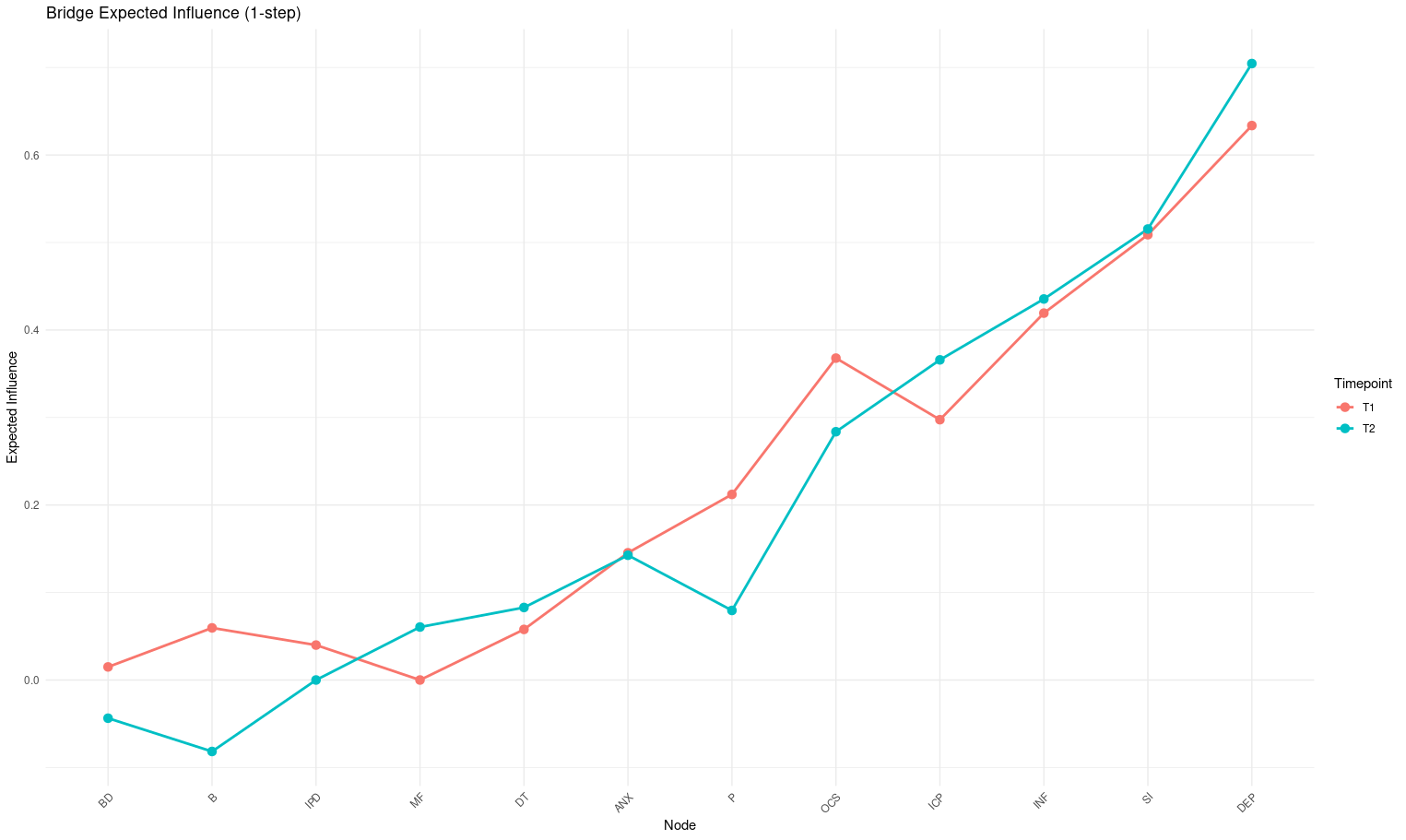


Figure S27

Bridge expected influence (1-step) for all nodes in the original network

*Note.* Values represent bridge expected influence (1-step), calculated as the sum of all signed (1-step) connections from each node to nodes outside its assigned symptom cluster. Higher values indicate greater connectivity across symptom clusters, suggesting a potential bridging role. BD, EDI-2 scale body dissatisfaction; B, EDI-2 scale bulimia; IPD, EDI-2 scale interpersonal distrust; MF, EDI-2 scale maturity fears; DT, EDI-2 scale drive for thinness; ANX, anxiety symptom score of SCL-90-R; P, EDI-2 scale perfectionism; OCS, obsessive-compulsive symptom score of SCL-90-R; ICP, EDI-2 scale interoception; INF, EDI-2 scale ineffectiveness; SI, suicidal ideation; DEP, depressive symptom score of SCL-90-R (generated without item #15).

References cited here:

Bargiacchi, A., Clarke, J., Paulsen, A., Leger, J., 2019. Refeeding in anorexia nervosa. *Eur. J.*

*Pediatr*. 178, 413–422. https://doi.org/10.1007/s00431-018-3295-7.

Bertoli, E., & De Leeuw, R. (2016). Prevalence of suicidal ideation, depression, and

anxiety in chronic temporomandibular disorder patients. *Journal of Oral & Facial Pain and Headache*, 296–301. https://doi.org/10.11607/ofph.1675

Desseilles, M., Perroud, N., Guillaume, S., Jaussent, I., Genty, C., Malafosse, A., & Courtet,

P. (2012). Is it valid to measure suicidal ideation by depression rating scales? *Journal of Affective Disorders*, 136(3), 398–404. https://doi.org/10.1016/j.jad.2011.11.013

Dunn, T. J., Baguley, T. & Brunsden, V. (2013). From alpha to omega: A practical solution to the pervasive problem of internal consistency estimation. *British Journal Of Psychology*, *105*(3), 399–412. https://doi.org/10.1111/bjop.12046

Favaro, A., & Santonastaso, P. (1997). Suicidality in eating disorders: Clinical and psycholo

gyical correlates. *Acta Psychiatrica Scandinavica*, *95*(6), 508–514. https://doi.org/10.1111/j.1600-0447.1997.tb10139.x

Fichter M, Quadflieg N. *Strukturiertes Inventar Für Anorektische Und Bulimische*

*Eßstörungen Nach DSM-IV Und ICD-10 (SIAB)*. 1st ed. Hogrefe; 1999.

Fichter M, Quadflieg N. The structured interview for anorexic and bulimic disorders for

DSM-IV and ICD-10 (SIAB-EX): reliability and validity. *Eur psychiatr*. 2001;16(1):38-48. doi:10.1016/S0924-9338(00)00534-4

Ganzeboom, H.B.G. De Graaf, P.M. & Treiman, D. J. (1992): A Standard International Socio-

Economic Index of Occupational Status. *Social Science Research 21* (1), 1-56.

Kämpfer, N., Staufenbiel, S., Wegener, I., Rambau, S., Urbach, A. S., Mücke, M., Geiser, F.,

& Conrad, R. (2016). Suicidality in patients with somatoform disorder – the speechless expression of anger? *Psychiatry Research*, *246*, 485–491. https://doi.org/10.1016/j.psychres.2016.10.022

Lazar C (2015). imputeLCMD: A collection of methods for left-censored missing data imputation. R package version 2.0. https://CRAN.R-project.org/package=imputeLCMD

McDonald, R. P. (2013). Test theory. In *Psychology Press eBooks*.
https://doi.org/10.4324/9781410601087

Meng, H., Li, J., Loerbroks, A., Wu, J., & Chen, H. (2013). Rural/urban Background,

Depression and Suicidal Ideation in Chinese College Students: A Cross-Sectional Study. *PLoS ONE*, *8*(8), e71313. https://doi.org/10.1371/journal.pone.0071313

Milos, G., Spindler, A., Hepp, U., & Schnyder, U. (2004). Suicide attempts and suicidal

ideation: links with psychiatric comorbidity in eating disorder subjects. *General Hospital Psychiatry*, *26*(2), 129–135. https://doi.org/10.1016/j.genhosppsych.2003.10.005

Miotto, P., De Coppi, M., Frezza, M., Petretto, D., Masala, C., & Preti, A. (2003).

Suicidal ideation and aggressiveness in school-aged youths. Psychiatry Research, 120(3), 247–255. https://doi.org/10.1016/s0165-1781(03)00193-8

Wei, R., Wang, J., Jia, E., Chen, T., Ni, Y., & Jia, W. (2018). GSimp: A Gibbs sampler based left-censored missing value imputation approach for metabolomics studies. *PLOS Computational Biology*. doi: 10.1371/journal.pcbi.1005973
